# Efficient synthesis of 1,2-disubstituted benzimidazoles catalyzed by phosphoric acid as a homogeneous catalyst under mild conditions and investigating their anti-diabetes properties through molecular docking studies and calculations†

**DOI:** 10.1039/d3ra07156a

**Published:** 2023-12-08

**Authors:** Azam Moazeni Bistgani, Abdulhamid Dehghani, Leila Moradi

**Affiliations:** a Department of Organic Chemistry, Faculty of Chemistry, University of Kashan Kashan P. O. Box 8731753153 Iran l_moradi@kashanu.ac.ir +98 3155912336

## Abstract

A green practical method for the efficient synthesis of 1-benzyl-2-phenyl-benzimidazole and its derivatives using phosphoric acid as an eco-friendly homogeneous catalyst from the condensation reaction of *o*-phenylenediamine (OPD) and aromatic aldehydes (bearing electron-withdrawing and electron-releasing groups) in methanol under thermal conditions is described. The advantages of this environmentally benign and safe protocol include short reaction times, very mild reaction conditions, excellent yields, not requiring specialized equipment, and simple workup procedures. This method obtained the desired products in moderate to excellent yields between 61–89% within a short period of time of about 13–30 minutes under mild reaction conditions. Finally, with the help of computational chemistry and drug design methods, the anti-diabetic properties of these compounds were studied and investigated. All the synthesized compounds bind to an agonist in the active site of the 4ll1 protein. These connections lead to the inactivation of this protein and create beneficial effects during the treatment of diabetes. In the synthesized compounds, one of the ligands establishes hydrogen bonds with glutamine 107 residues through nitrogens, and in addition, it establishes Π bonds with tyrosine 72. In this study, it was found that these compounds have the potential to become an oral anti-diabetic drug.

## Abstract 2

A green practical method for the efficient synthesis of 1-benzyl-2-phenyl-benzimidazole and its derivatives using phosphoric acid as an eco-friendly homogeneous catalyst from the condensation reaction of *o*-phenylenediamine (OPD) and aromatic aldehydes (bearing electron-withdrawing and electron-releasing groups) in methanol under thermal conditions is described.

## Graphical abstract

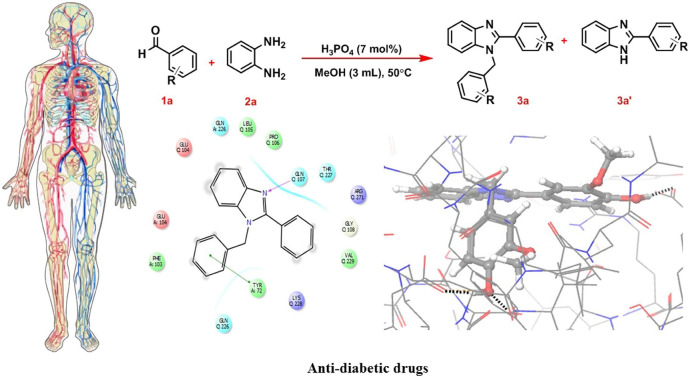

## Introduction

**Fig. 1:**
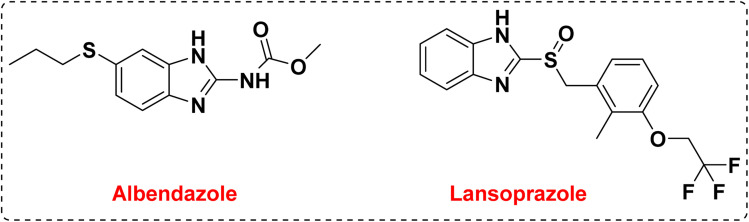
Two common drugs in the treatment of diabetes with benzimidazole structural skeleton.

One of the goals of medicinal chemistry is to design, prepare, and characterize the mode of action of bioactive compounds at the molecular level, which have high value and properties in the treatment and improvement of diseases.^1–4^ Among these active compounds, heterocycle compounds are a group of organic compounds that are of particular importance in the discovery of life, the discovery of drugs, and the improvement of their properties.^5–8^ Heterocycle compounds play an important role in the survival, reproduction, and evolution of life in molecular forms which is why researchers are trying to design and synthesize drugs based on heterocycles. In this regard, heterocycles containing nitrogen heteroatoms are among the compounds that have received special attention in medicinal chemistry.^9–16^ Benzimidazoles and their derivatives are considered to be the most important heterocycle compounds containing nitrogen heteroatoms, which are formed by the fusion of benzene and imidazole as an important pharmacophore.^17–20^ They can be prepared using various reactants to form the C–N bonds such as the reaction of *o*-phenylene diamine with benzaldehydes or primary alcohols, or by the reaction of *o*-nitro aniline and benzaldehydes, benzyl alcohol or CO_2_.^21^ The benzimidazole system is one of the components and core of many drugs and natural vitamins such as vitamin B12. The structural skeleton of benzimidazoles is found in many pharmaceutical compounds.^22–25^ Drugs with a benzimidazole skeleton include anthelmintic drugs albendazole, fenbendazole, oxfenbendazole, thiabendazole, and mebendazole, and proton pump inhibitors omeprazole, lansoprazole and pantoprazole. The most important medicinal properties of these compounds are anti-hypertensive, anti-parasitic, anti-worm, anti-HIV, anti-seizure, anti-diabetes, anti-inflammatory, anti-neoplastic, anti-trichinosis, and treatment of nematode and trematode infections.^26–31^ Also, benzimidazoles are used in many organic chemistry reactions, and in other fields such as agriculture, electronics and polymer chemistry.^32–34^ The preparation of this group of compounds enables an efficient and useful method for the synthesis of small and large organic compounds that are biologically effective and speed up the synthesis process of new drugs.^35–37^ Currently, nitrogen-containing heterocyclic compounds containing the benzimidazole core have attracted the attention of researchers in search of new agents with specific medicinal properties. Among the different derivatives of these compounds, the preparation of 1,2-disubstituted benzimidazole derivatives has received much attention from chemists in terms of biological properties and medicinal applications (Fig. 1).

**Fig. 2:**
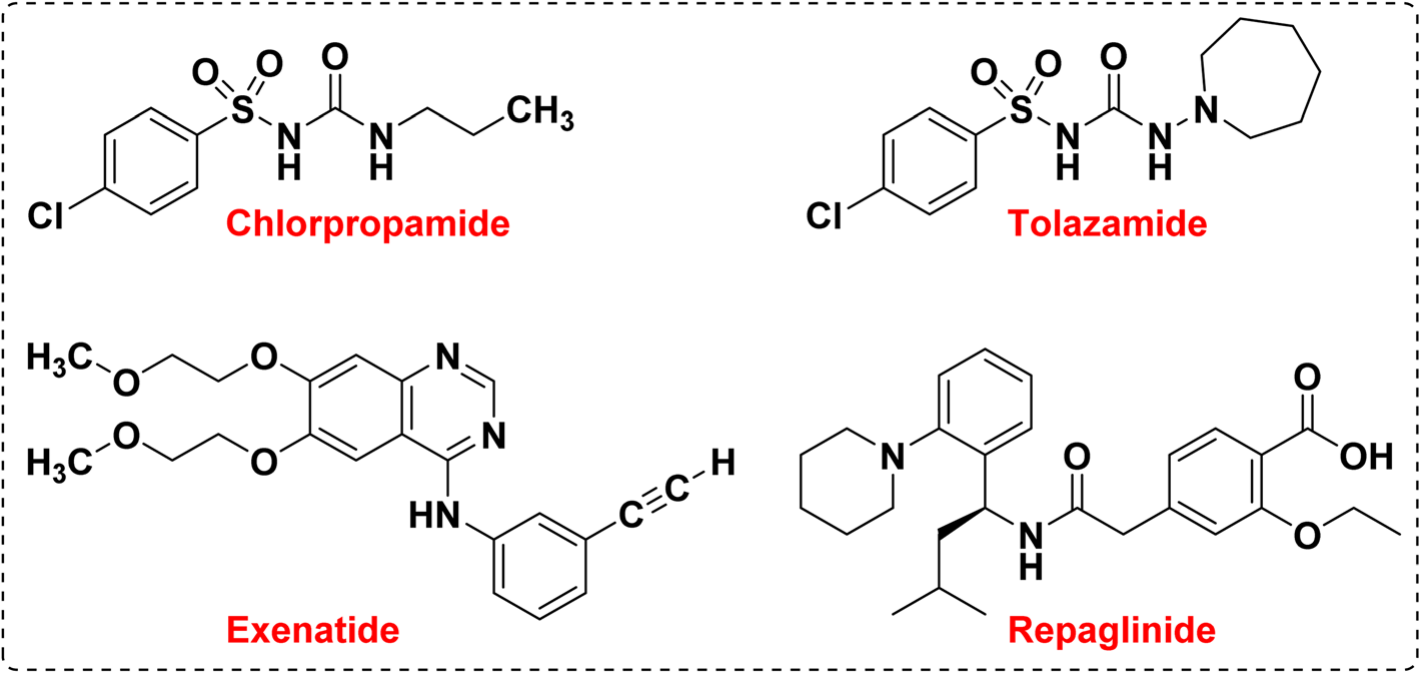
Examples of common agents in the treatment and control of diabetes.

Diabetes is a chronic metabolic disease that is divided into three categories: type I, type II, and pregnancy.^38,39^ Type I diabetes is defined as insulin-dependent diabetes, which is caused by autosomal recessive and X-linked recessive mutations. In this type of diabetes, the cellular immune reaction causes the destruction of beta cells in the pancreas, which causes hyperglycemia with the loss of 80–90% of these cells. This type of diabetes accounts for about 5–10% of all types of diabetes. Symptoms of type I diabetes include dry mouth and excessive thirst, blurred vision, frequent urination, and sudden feeling of fatigue.^40–43^ But type II diabetes, which is defined as non-insulin-dependent diabetes, accounts for 90–95% of all types of diabetes. In this type of diabetes, cell receptors in the target organs are resistant to insulin and hyperglycemia develops gradually, in other words, at the beginning of the disease, insulin deficiency is relative, however, patients are exposed to macrovascular and microvascular factors. Non-insulin-dependent diabetes depends on factors such as inactivity, the aging population, and increasing obesity.^44–47^ Symptoms of type II diabetes include urinary infections, delayed healing of wounds and cuts, impotence, blurred vision, and tingling in the hands and feet. Antidiabetic drugs are used to lower blood glucose in people with diabetes, and these drugs are divided into groups that stimulate glucose excretion and insulin secretion, reduce insulin resistance, and peptide analogs.^48–52^ In the last few decades, there have been reports on the design and synthesis of new medicinal compounds in the control and treatment of diabetes (Fig. 2).

**Scheme 1:**
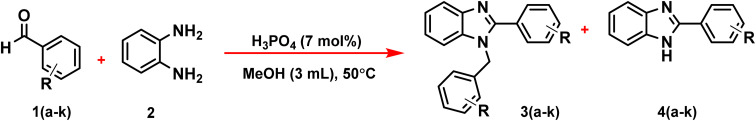
Synthesis of 1,2-disubstituted benzimidazoles (3a–k), and 1-substituted benzimidazoles (4a–k) in the presence of phosphoric acid as a catalyst.

In this research, the authors try to investigate the effect of phosphoric acid as an effective catalyst in the solvent of methanol in the synthesis of benzimidazoles by aryl aldehydes with *ortho* phenylenediamine with high efficiency and short reaction time. In addition, the antidiabetic properties of these compounds were investigated through molecular docking calculations (Scheme 1).

## Experimental section

### Instrumentation, analyses, and starting material

All substrates and solvents were purchased from Fluka, Aldrich, and Merck chemical companies used without purification. The reaction monitoring was accomplished by thin layer chromatography (TLC) on silica-gel plates (Merck 60 F 250) containing a fluorescence indicator (*λ* = 254 nm). ^1^H NMR and ^13^C NMR spectra were recorded on a Bruker DRX Avance spectrometer (400 and 100 MHz, respectively) using pure deuterated DMSO-d_6_ as solvent and tetramethylsilane as internal standard. Chemical shifts are given in the *δ* scale in part per million (ppm) and coupling constants in Hertz the following abbreviations are used for the multiplicities: s = singlet, d = doublet, dd = doublet of doublet, t = triplet, m = multiplet, signal for proton spectra. Melting points were measured in capillary tubes in a Scientific Thermo apparatus (model 9300, England). The mode of interaction was investigated by docking. The ligand–receptor interaction pictures were created using Schrödinger 2018.10 software.

### General procedure for the preparation of 1,2-disubstituted benzimidazoles derivatives

To a 50 mL round-bottom flask containing methanol (3 mL), benzaldehyde 1 (2 mmol), *o*-phenylenediamine 2 (1 mmol), and phosphoric acid (7 mol%) were added and the resulting mixture was stirred magnetically at 50 °C for 5 minutes. The progress of the reactions was probed by thin layer chromatography (*n*-hexane/EtOAc 6 : 4). Then the reaction mixture was diluted with water and centrifuged to remove the catalyst. The filtrate was extracted with CH_2_Cl_2_ and water. The organic layer was dried with MgSO_4_. The residue was purified by column chromatography on silica gel (*n*-hexane/ethyl acetate 12 : 8) to afford the desired pure product. The pure products are ready for further characterization. The structure of the synthesized compounds was investigated by spectroscopic methods such as NMR (^1^H and ^13^C), and computational docking investigations were performed on their structures by Schrödinger 2015 software (Maestro 10.2). Spectral information related to the prepared products is given below.

### Spectral data (compound 3a–3k)

#### 1-Benzyl-2-phenyl-1*H*-benzo[*d*]imidazole (compound 3a)

Yield: 255 mg (90%); white solid; ^1^H NMR (400 MHz, DMSO-d_6_): *δ* (ppm) = 7.76–7.73 (m, 2H, ArH), 7.56–7.46 (m, 5H, ArH), 7.29–7.23 (m, 5H, ArH), 7.02–7.00 (m, 2H, ArH), 5.59 (s, 1H, CH_2_); ^13^C NMR (101 MHz, DMSO-d_6_): *δ* (ppm) = 153.2, 142.7, 136.9, 135.8, 129.8, 129.0, 128.9, 128.7, 127.4, 126.4, 126.0, 122.6, 122.2, 119.2, 111.1, 47.4.

#### 1-(3-Nitrobenzyl)-2-(3-nitrophenyl)-1*H*-benzo[*d*]imidazole (compound 3b)

Yield: 329 mg (88%); yellow solid; ^1^H NMR (400 MHz, DMSO-d_6_): *δ* (ppm) = 8.62 (t, *J* = 4.0 Hz, 1H, ArH), 8.54–8.51 (m, 1H), 8.35–8.33 (m, 1H, ArH), 8.30–8.27 (m, 1H, ArH), 8.12 (s, 1H, ArH), 8.00–7.95 (m, 2H, ArH), 7.81–7.73 (m, 2H, ArH), 7.57 (d, *J* = 8.0 Hz, 1H, ArH), 7.51–7.48 (m, 2H, ArH), 5.98 (s, 2H, CH_2_); ^13^C NMR (101 MHz, DMSO-d_6_): *δ* (ppm) = 150.9, 147.8, 142.5, 139.1, 136.0, 135.3, 132.7, 131.2, 130.6, 130.5, 124.5, 123.7, 123.5, 122.8, 122.5, 121.3, 119.7, 111.2, 46.8.

#### 1-(4-Methoxybenzyl)-2-(4-methoxyphenyl)-1*H*-benzo[*d*]imidazole (compound 3c)

Yield: 271 mg (79%); white solid; ^1^H NMR (400 MHz, DMSO-d_6_): *δ* (ppm) = 7.70–7.67 (m, 3H, ArH), 7.46–7.43 (m, 1H, ArH), 7.24–7.20 (m, 2H, ArH), 7.11–7.08 (m, 2H, ArH), 6.95 (d, *J* = 8.0 Hz, 2H, ArH), 6.85 (d, *J* = 8.0 Hz, 2H, ArH), 5.50 (s, 2H, CH_2_), 3.82 (s, 3H, OMe), 3.68 (s, 3H, OMe); ^13^C NMR (101 MHz, DMSO-d_6_): *δ* (ppm) = 160.3, 158.5, 153.1, 142.7, 135.8, 130.5, 128.8, 127.9, 127.4, 122.3, 122.3, 122.0, 121.5, 118.9, 114.3, 114.2, 114.1, 110.9, 55.3, 55.0, 46.9.

#### 1-(3,4-Dimethoxybenzyl)-2-(3,4-dimethoxyphenyl)-1*H*-benzo[*d*]imidazole (compound 3d)

Yield: 298 mg (74%); white solid; ^1^H NMR (400 MHz, DMSO-d_6_): *δ* (ppm) = 7.87–7.82 (m, 1H, ArH), 7.66–7.61 (m, 1H, ArH), 7.44–7.42 (m, 2H, ArH), 7.40–7.34 (m, 2H, ArH), 7.25 (d, *J* = 8.0 Hz, 1H, ArH), 6.98 (d, *J* = 8.0 Hz, 1H, ArH), 6.90 (d, *J* = 1.9 Hz, 1H, ArH), 6.59 (dd, *J* = 8.3, 1.9 Hz, 1H, ArH), 5.64 (s, 2H, CH_2_), 3.96 (s, 3H, OMe), 3.86 (s, 3H, OMe), 3.81 (s, 3H, OMe), 3.78 (s, 3H, OMe); ^13^C NMR (101 MHz, DMSO-d_6_): *δ* (ppm) = 153.2, 150.0, 148.8, 148.6, 148.0, 142.6, 136.0, 129.3, 122.4, 122.3, 122.0, 121.6, 118.9, 118.0, 112.4, 111.8, 111.6, 110.9, 110.1, 55.6, 55.4, 55.4, 55.3.

#### 1-(4-Methylbenzyl)-2-(*p*-tolyl)-1*H*-benzo[*d*]imidazole (compound 3e)

Yield: 252 mg (81%); white solid; ^1^H NMR (400 MHz, DMSO-d_6_): *δ* (ppm) = 7.74–7.72 (m, 1H, ArH), 7.63 (d, *J* = 8.0 Hz, 2H, ArH), 7.42–7.41 (m, 1H, ArH), 7.32 (d, *J* = 8.0 Hz, 2H, ArH), 7.25–7.21 (m, 2H, ArH), 7.08 (d, *J* = 8.0 Hz, 2H, ArH), 6.90 (d, *J* = 8.0 Hz, 2H, ArH), 5.51 (s, 2H, CH_2_), 2.36 (s, 3H, Me), 2.22 (s, 3H, Me); ^13^C NMR (101 MHz, DMSO-d_6_): *δ* (ppm) = 153.3, 142.7, 139.5, 136.6, 135.8, 133.9, 129.5, 129.3, 128.9, 127.3, 126.4, 125.9, 122.4, 122.1, 119.1, 111.0, 47.2, 20.9, 20.5.

#### 3-(1-(3-Hydroxybenzyl)-1*H*-benzo[*d*]imidazole-2-yl)phenol (compound 3f)

Yield: 233 mg (74%); pink solid; ^1^H NMR (400 MHz, DMSO-d_6_): *δ* (ppm) = 9.83 (s, 1H, OH), 9.46 (s, 1H, OH), 7.74–7.72 (m, 1H, ArH), 7.41–7.39 (m, 1H, ArH), 7.32 (t, *J* = 8.0 Hz, 1H), 7.28–7.21 (m, 2H, ArH), 7.19–7.17 (m, 1H, ArH), 7.15–7.09 (m, 2H, ArH), 6.96–6.93 (m, 1H, ArH), 6.65 (dd, *J* = 8.0, 1.7 Hz, 1H, ArH), 6.49 (d, *J* = 4.0 Hz, 1H, ArH), 6.40 (d, *J* = 4.0 Hz, 1H, ArH), 5.49 (s, 2H, CH_2_).; ^13^C NMR (101 MHz, DMSO-d_6_): *δ* (ppm) = 157.7, 157.5, 153.3, 142.6, 138.3, 135.8, 131.2, 129.8, 122.6, 122.1, 119.4, 119.2, 116.9, 116.6, 116.0, 114.4, 112.6, 111.1, 47.4.

#### 4-(1-(4-Hydroxybenzyl)-1*H*-benzo[*d*]imidazole-2-yl)phenol (compound 3g)

Yield: 233 mg (74%); white solid; ^1^H NMR (400 MHz, DMSO-d_6_): *δ* (ppm) = 9.98 (s, 1H, OH), 9.42 (s, 1H, OH), 7.67–7.65 (m, 1H, ArH), 7.59–7.59 (m, 2H, ArH), 7.43–7.41 (m, 1H, ArH), 7.23–7.15 (m, 2H, ArH), 6.92–6.89 (m, 2H, ArH), 6.84 (d, *J* = 8.0 Hz, 2H, ArH), 6.69–6.65 (m, 2H, ArH), 5.42 (s, 2H, CH_2_); ^13^C NMR (101 MHz, DMSO-d_6_): *δ* (ppm) = 160.1, 157.9, 154.8, 144.0, 137.1, 131.9, 128.8, 128.4, 123.4, 123.1, 122.1, 120.1, 116.8, 116.7, 112.2, 48.3.

#### 5-(1-(3-Hydroxy-4-methoxybenzyl)-1*H*-benzo[*d*]imidazole-2-yl)-2-methoxyphenol (compound 3h)

Yield: 229 mg (61%); white solid; ^1^H NMR (400 MHz, DMSO-d_6_: *δ* (ppm) = 9.40 (s, 1H, OH), 9.05 (s, 1H, OH), 7.69–7.67 (m, 1H, ArH), 7.38–7.36 (m, 1H, ArH), 7.24–7.17 (m, 3H, ArH), 7.13 (dd, *J* = 8.3, 2.1 Hz, 1H, ArH), 7.05 (d, *J* = 8.3 Hz, 1H, ArH), 6.84 (d, *J* = 8.0 Hz, 1H, ArH), 6.47–6.43 (m, 2H, ArH), 5.42 (s, 2H, CH_2_), 3.83 (s, 3H, OMe), 3.71 (s, 3H, OMe); ^13^C NMR (101 MHz, DMSO-d_6_): *δ* (ppm) = 153.3, 149.1, 146.9, 146.7, 146.5, 142.6, 135.9, 129.4, 122.5, 122.2, 121.9, 120.1, 118.9, 116.7, 116.2, 113.2, 112.3, 111.9, 110.9, 55.6, 55.5, 47.0.

#### 4-(1-(4-Hydroxy-3-methoxybenzyl)-1*H*-benzo[*d*]imidazole-2-yl)-2-methoxyphenol (compound 3i)

Yield: 236 mg (63%); yellow solid; ^1^H NMR (400 MHz, DMSO-d_6_): *δ* (ppm) = 9.58 (s, 1H, OH), 9.00 (s, 1H, OH), 7.69–7.66 (m, 1H, ArH), 7.50–7.48 (m, 1H, ArH), 7.26–7.18 (m, 4H, ArH), 6.92 (d, *J* = 8.0 Hz, 1H, ArH), 6.70–6.65 (m 2H), 6.38 (dd, *J* = 8.0, 4.0 Hz, 1H, ArH), 5.45 (s, 1H, CH_2_), 3.72 (s, 1H, OMe), 3.64 (s, 1H, OMe); ^13^C NMR (101 MHz, DMSO-d_6_): *δ* (ppm) = 153.5, 148.1, 147.6, 147.5, 145.8, 142.6, 136.0, 127.8, 122.1, 122.0, 121.9, 121.1, 118.8, 118.5, 115.5, 115.5, 112.9, 110.8, 110.6, 55.4, 55.4, 47.3.

#### 1-(4-Chlorobenzyl)-2-(4-chlorophenyl)-1*H*-benzo[*d*]imidazole (compound 3j)

Yield: 314 mg (89%); white solid; ^1^H NMR (400 MHz, DMSO-d_6_): *δ* (ppm) = 7.77–7.72 (m, 3H, ArH), 7.62–7.59 (m, 2H, ArH), 7.52–7.48 (m, 1H, ArH), 7.38–7.34 (m, 2H, ArH), 7.29–7.26 (m, 2H, ArH), 7.01 (d, *J* = 8.0 Hz, 2H, ArH), 5.60 (s, 2H, CH_2_); ^13^C NMR (101 MHz, DMSO-d_6_): *δ* (ppm) = 152.0, 142.6, 135.8, 134.7, 132.1, 129.0, 128.9, 128.8, 128.8, 128.1, 128.0, 123.0, 122.4, 119.4, 111.1, 46.8.

#### 2-(Thiophen-2-yl)-1-(thiophen-2-ylmethyl)-1*H*-benzo[*d*]imidazole (compound 3k)

Yield: 248 mg (84%); white solid; ^1^H NMR (400 MHz, DMSO-d_6_): *δ* (ppm) = 7.85–7.82 (m, 1H, ArH), 7.75–7.69 (m, 3H, ArH), 7.44–7.38 (m, 1H, ArH), 7.28–7.24 (m, 3H, ArH), 7.04–7.03 (m, 1H, ArH), 6.98–6.94 (m, 1H, ArH), 5.95 (s, CH_2_); ^13^C NMR (101 MHz, DMSO-d_6_): *δ* (ppm) = 142.4, 135.8, 129.7, 128.4, 127.8, 127.0, 126.1, 126.0, 122.6, 118.9, 110.8, 43.0.

## Results and discussion

### Screening the conditions for the synthesis of 1,2-disubstituted benzimidazoles in the presence of phosphoric acid

In order to establish the optimum conditions, the catalytic activities of various acid was examined in a model reaction using benzaldehyde and *o*-phenylenediamine. Initially, the effect of acid, on the model reaction was investigated. Because of the critical role of acid in reaction, the effectiveness of various acids such as acetic acid, trichloroacetic acid, *p*-toluenesulfonic acid, chromotropic acid, meglumine sulfate, phosphoric acid, AlCl_3_, BF_3_·Et_2_O, and [Bmim]BF_4_, were studied in the model reaction. Although all of the acids applied showed good activity (Table 1, entries 1–10) the most effective acid was phosphoric acid (Table 1, entry 6). Afterward, for choosing the reaction media, different solvents such as EtOH, MeOH, H_2_O, DMF, and water and EtOH mixture (1 : 1) were examined (Table 1 and entries 11–14) and the best results were obtained in the MeOH (Table 1 and entry 13). In the next step, the amount of the catalyst on the reaction rate was investigated. Then, 3, 5, 7, and 10 mol% of catalyst were used in the model reaction (Table 1 and entry 13, entries 15–17). When we used 7 mol% of catalyst, the highest efficiency in the product was observed (Table 1 and entry 16). When 10 mol% of catalyst was used, the reaction efficiency decreased, even when 3 and 5 mol% of catalyst was used, the reaction efficiency decreased slightly (Table 1, entries 13, 15, and 17). In order to measure the effect of temperature on reaction efficiency and reaction time, the reaction was studied at four temperatures r.t., 40 °C, 50 °C, and 60 °C (Table 1, entry 16 and entries 18–20). The best efficiency was observed when the reaction temperature was 50 °C, whereas, increasing the temperature to more than 50 °C, led to a decrease in the yield (Table 1, entry 20). Thereupon, the optimized conditions were found to be using methanol as solvent, in the presence of 7 mol% of catalyst (phosphoric acid), 50 °C, and 5 min reaction time (Table 1, entry 16).

**Table 1:** Optimization of the reaction conditions for the synthesis of 1-benzyl-2-phenyl-1*H*-benzo[*d*]imidazole (3a) and 2-phenyl-1*H*-benzo[*d*]imidazole (4a)^a^

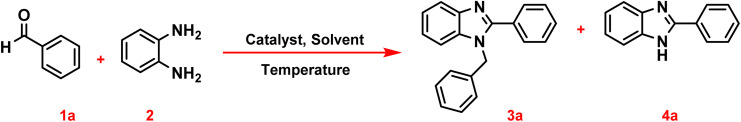					
Entry	Solvent	Catalyst	Temp. (°C)	Time (min)	Yield (3a : 4a)^b^
1	EtOH	Acetic acid (3 mol%)	50	25	25 : 6
2	EtOH	Trichloroacetic acid (3 mol%)	50	25	30 : 4
3	EtOH	*P*-toluenesulfonic acid (3 mol%)	50	25	—
4	EtOH	Chromotropic acid (3 mol%)	50	25	20 : 5
5	EtOH	Meglumine sulfate (3 mol%)	50	25	35 : 4
6	EtOH	Phosphoric acid (3 mol%)	50	25	48 : 6
7	EtOH	AlCl_3_ (3 mol%)	50	25	20 : 7
8	EtOH	BF_3_·Et_2_O (3 mol%)	50	25	20 : 6
9	EtOH	[Bmim]BF_4_ (3 mol%)	50	25	25 : 4
10	H_2_O	Meglumine sulfate (3 mol%)	50	25	35 : 6
11	H_2_O	Phosphoric acid (3 mol%)	50	25	27 : 5
12	H_2_O/EtOH (1 : 1)	Phosphoric acid (3 mol%)	50	25	32 : 6
13	MeOH	Phosphoric acid (3 mol%)	50	25	56 : 4
14	DMF	Phosphoric acid (3 mol%)	50	25	25 : 6
15	MeOH	Phosphoric acid (5 mol%)	50	20	73 : 5
**16**	**MeOH**	**Phosphoric acid (7 mol%)**	**50**	**15**	**90 : 5** c
17	MeOH	Phosphoric acid (10 mol%)	50	15	58 : 6
18	MeOH	Phosphoric acid (7 mol%)	r.t.	20	54 : 5
19	MeOH	Phosphoric acid (7 mol%)	40	20	78 : 5
20	MeOH	Phosphoric acid (7 mol%)	60	20	63 : 4

a Reaction conditions: benzaldehyde (2 mmol) and *o*-phenylenediamine (1 mmol), catalyst and solvent (3 mL).

b TLC yield.

c Isolated yield.

Encouraged by the initial success in the production of 1-benzyl-2-phenyl-1*H*-benzo[*d*]imidazole (3a) *via* the condensation reaction strategy, to show the general scope and versatility of this strategy in the preparation of 1,2-disubstituted benzimidazoles, different substituted aromatic aldehydes, and *o*-phenylenediamine were examined under optimized conditions. Excitingly, the corresponding 1,2-disubstituted benzimidazole derivatives were successfully and smoothly obtained, and the results are listed in Table 2. As seen from Table 2, aromatic aldehydes with electron-withdrawing groups such as NO_2_ reacted faster than those with electron-releasing such as OCH_3_, CH_3_, and OH.

**Table 2:** Synthesis of 1,2-disubstituted benzimidazole derivatives catalyzed by phosphoric acid^a^

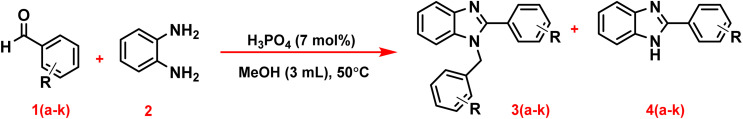						
Entry	R	Product	Time (min)	Yield 3 : 4 b (%)	m.p	Lit. m.p
1	H	3a	15	90 : 5	133–135	132–134 (ref. 53)
2	3-NO_2_	3b	13	88 : 9	154–156	154–155 (ref. 54)
3	4-OCH_3_	3c	15	79 : 6	126–128	127–129 (ref. 55)
4	3,4-di OCH_3_	3d	23	74 : 5	171–173	170–172 (ref. 56)
5	4-CH_3_	3e	15	81 : 7	128–130	126–129 (ref. 57)
6	3-OH	3f	15	74 : 7	257–258	250–252 (ref. 58)
7	4-OH	3g	15	74 : 9	208–210	206–208 (ref. 59)
8	3-OH, 4-OCH_3_	3h	30	61 : 14	228–230	229–231 (ref. 60)
9	3-OCH_3_, 4-OH	3i	30	63 : 12	184–186	187–189 (ref. 61)
10	4-Cl	3j	15	89 : 6	131–133	131–134 (ref. 62)
11	Thiophene-2-carbaldehyde	3k	20	84 : 8	146–149	146–149 (ref. 63)

a Reaction condition: different aromatic aldehyde (2 mmol), *o*-phenylenediamine (1 mmol), phosphoric acid (7 mol%), MeOH (3 mL), 50 °C.

b Isolated yield.

### Mechanism of the catalytic reaction

**Scheme 2:**
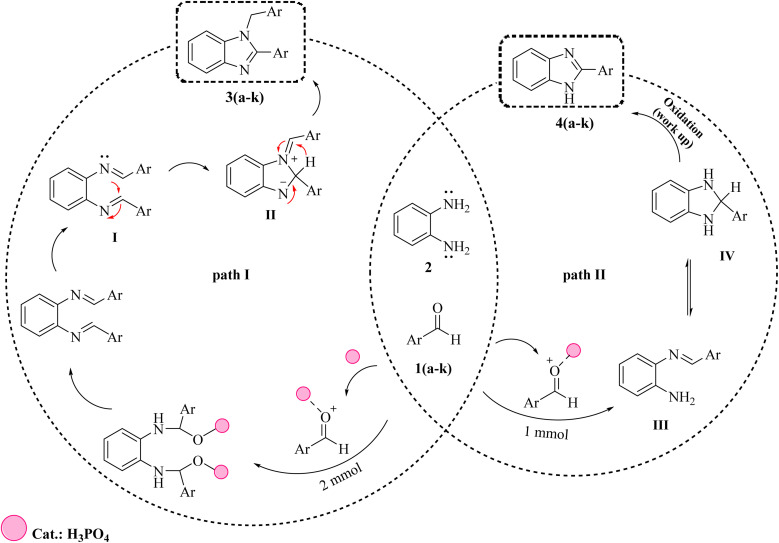
Plausible reaction mechanism for the formation of 1,2-disubstituted benzimidazoles (3a–k) and 1-substituted benzimidazoles (4a–k).

**Fig. 3:**
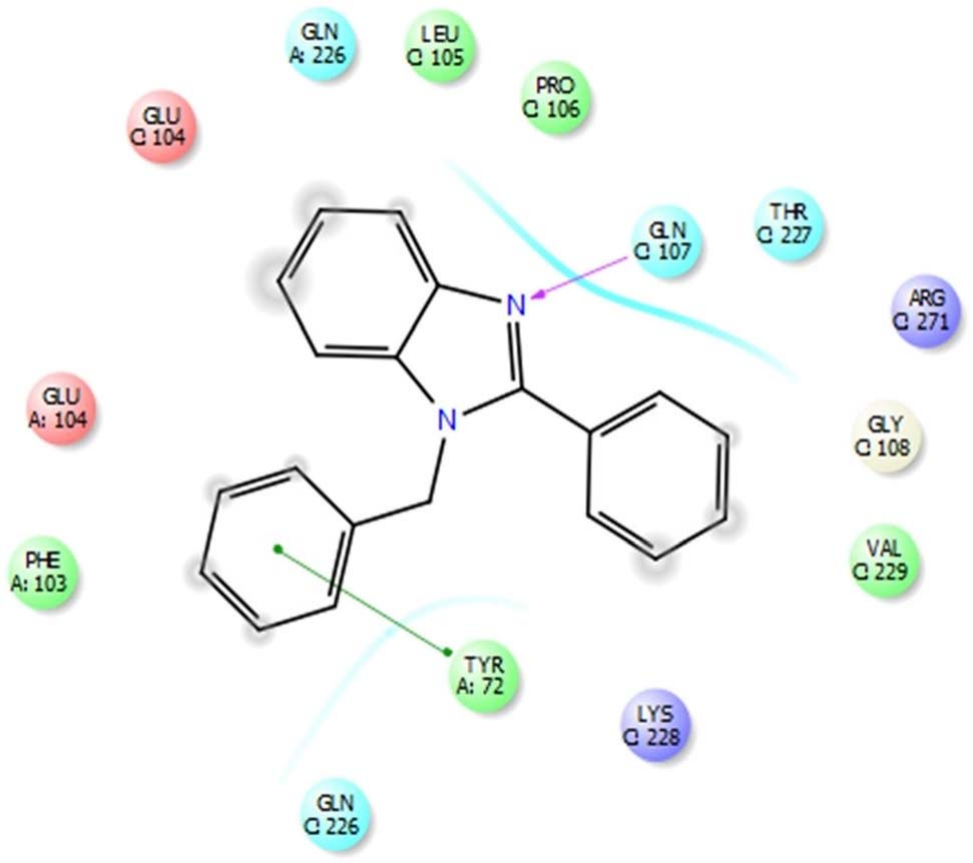
Bonds and interactions between ligand 3a and protein 4ll1.

Scheme 2 illustrates possible mechanisms for the formation of 1,2-disubstituted benzimidazoles (3a–k), and 1-substituted benzimidazoles (4a–k).^64^ As can be seen, initially phosphoric acid catalyst accelerates the nucleophilic attack of *ortho* phenylenediamine (2) by activating aromatic carbonyl aldehydes (1a–k) and leads to the formation of bisimine intermediate (I) and monoimine intermediate (III). In the first pathway, after the formation of bisimine intermediate, due to the intermolecular nucleophilic attack, intermediate (I) is converted to intermediate (II). After that, the final product (3a–k) is formed due to a 1,3-hydrogen shift. In the second pathway, after the formation of the intermediate monoimine, the intermolecular nucleophilic attack leads to the formation of the imidazoline intermediate (IV). Finally, oxidation of intermediate (IV) leads to the formation of product 4a–k (Fig. 3).

### Molecular docking calculations of synthesized compounds

The results of molecular docking calculations of the synthesized compounds are given in Table 3.

**Table 3:** Results of molecular docking calculations for 3a–k compounds as ligand of protein 4ll1

Entry	Molecular weight	Octanol/water ratio	Number of acceptor hydrogen bonds	Number of donor hydrogen bonds	Cell permeability	Edibility percentage	Edible potential	Ligand-protein complex energy
3a	284.36	5.284	1.5	0	6529.465	84.723	3	−2.444
3b	374.355	3.82	3.5	0	96.868	100	3	−3.187
3c	344.412	5.437	3	0	6538.604	100	3	−4.749
3d	404.465	5.178	4.5	0	6497.772	100	3	−4.255
3e	312.413	5.945	1.5	0	6537.54	100	1	−3.726
3f	316.359	3.734	3	2	602.919	100	3	−4.512
3g	316.359	3.734	3	2	602.919	100	3	−4.512
3h	376.411	4.083	4.5	2	713.742	100	3	−4.305
3i	376.411	3.838	4.5	2	746.221	100	3	−5.315
3j	353.25	6.301	1.5	0	6542.484	100	3	−4.238
3k	296.404	5.061	1.5	0	6159.06	100	3	−3.188

Docking energy indicates the strength of the binding of the ligand to the receptor, the more negative the number is, the better the binding of the ligand to the receptor. The results of the analysis of molecular docking calculations according to Lee Pinsky's rules are given below.

#### Molecular mass

In this law, the molecular mass of the drug should not be more than 500 g mol^−1^, because the heavier the molecule becomes, the possibility of its absorption and permeability decreases. Fortunately, all synthesized compounds follow this law.

#### Ligand dissociation factor

This will create a balance between hydrophilicity and lipophilicity of the drug molecule. In this balance, the octanol/water partition coefficient should not exceed 5. This is not the case for 6 ligands (3a, 3c, 3d, 3e, 3j, and 3k with ligand dissociation factor of 5.284, 5.437, 5.178, 5.945, 6.301, 5.061 respectively).

#### Number of hydrogen donating groups

This item indicates the number of hydrogen donating groups (such as NH and OH) in the drug molecule. The number of these groups should not be more than 5, which according to the results, all combinations follow this case.

#### Number of hydrogen acceptor groups

This item indicates the number of hydrogen acceptor groups (such as N and O) in the drug molecule. The number of these groups should not be more than 5, which all combinations follow this case.

#### Cell permeability

This item plays an important role in bioavailability and drug absorption. Cell permeability optimizes the gastrointestinal absorption of drugs, which should have a permeability rate greater than 500 nm s^−1^. All combinations except 3b follow this case.

#### Edible potential

**Fig. 4:**
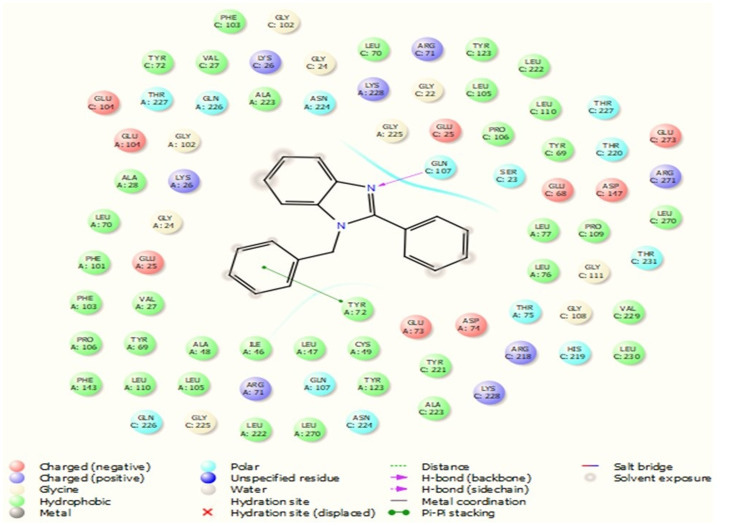
Links and connections of ligand 3a and extended protein 4ll1.

The potential of being edible is high for all ligands, except for two compounds 3e and 3j, whose edible potential is very low, as a result, they have very little absorption potential. The absorption percentage of other ligands in the body is high and 100% of the drug is absorbed (Fig. 4).

PHOA = prediction of drug absorption on a scale of 0 to 100 percent.

### Investigating and how protein 4ll1 binds to its natural ligand in the treatment of diabetes

**Fig. 5:**
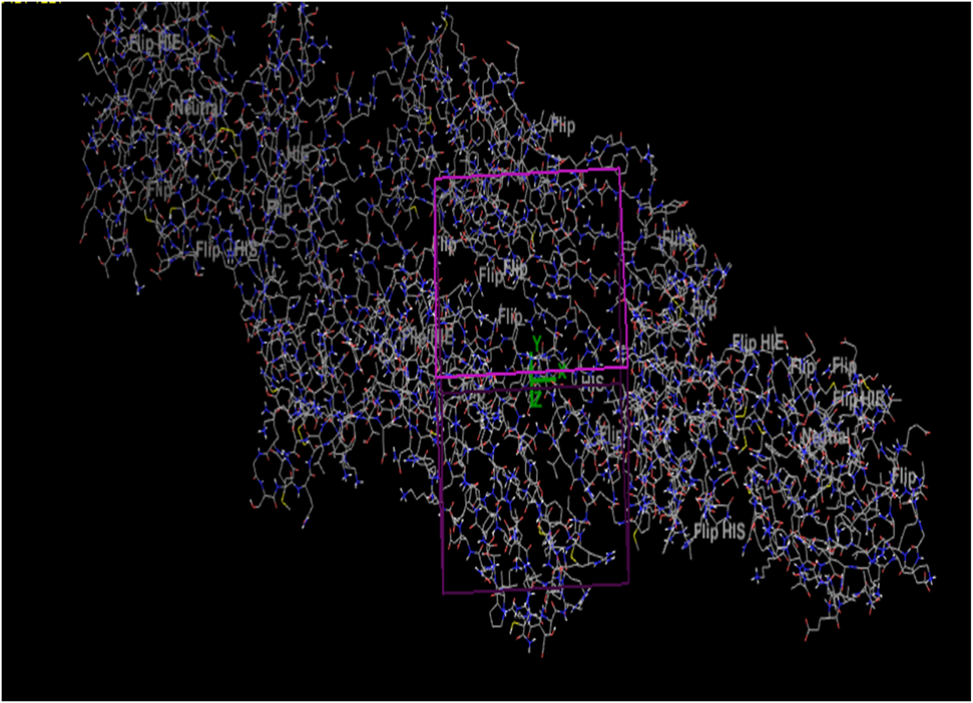
Active site of protein 4ll1.

This protein named thioredoxine-interacting (Txnip) is affected by both type I and type II diabetes. In pictures 3 and 4, links and connections of ligand with protein are shown in two dimensions. As shown in the figure, one of the ligands establishes hydrogen bonds with glutamine 107 residues through nitrogens, and in addition, it establishes Π bonds with tyrosine 72. These bonds play a very special and vital role in biological sciences and pharmaceutical compounds (Fig. 5).

**Fig. 6:**
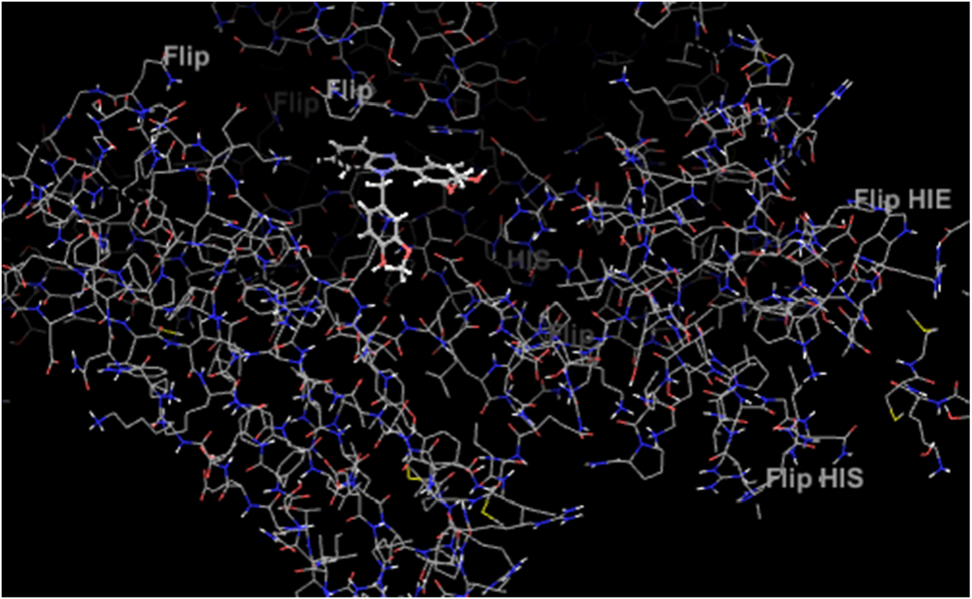
Molecular docking of derivatives on ligand 3i of protein 4ll1.

**Fig. 7:**
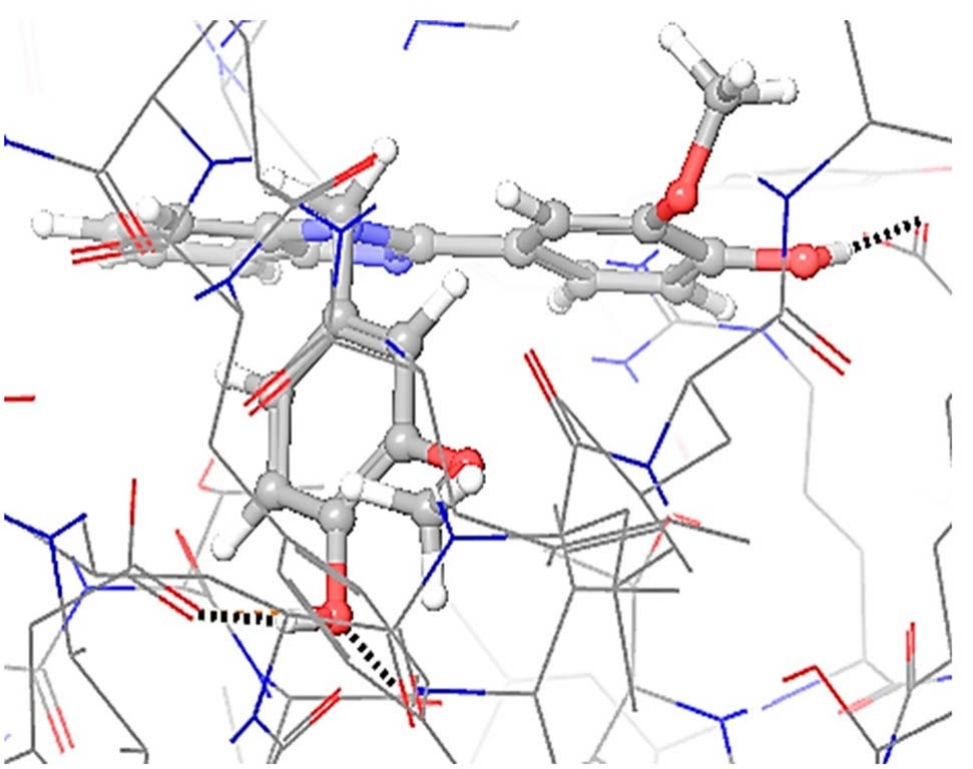
The interaction of ligand 3i and protein.

As shown in Fig. 6, all the synthesized compounds are attached to an agonist in the active site of the 4ll1 protein. These connections lead to the inactivation of this protein and create beneficial effects during the treatment of diabetes (Fig. 7).

To ascertain the capability of the applied homogeneous catalyst, the application to accomplish the consideration of the reaction between benzaldehyde and *ortho* phenylenediamine (OPD) was selected to be compared with the literature reports (Table 4). As can be observed in Table 4, the most of catalysts published catalyze the consideration of reaction to prepare 1-benzyl-2-phenyl-1*H*-benzoimidazole at higher temperatures, and using larger amounts of catalysts after longer reaction times in lower yields (Table 4, entry 1–21).

**Table 4:** Comparison among efficiency of phosphoric acid with the reported catalysts for the preparation of 1-benzyl-2-phenyl-1*H*-benzo[*d*]imidazole (3a) and 2-phenyl-1*H*-benzo[*d*]imidazole (4a)^a^

Entry	Catalyst	Conditions	Time (h)	Yield 3a : 4a^b^ (%)	Ref.
1	Scandium tris(trifluoromethanesulfonat)	Oxygen, tetrahydrofuran, 20 °C	44	1 : 97	63
2	Thiamine hydrochloride	DMF, 20 °C	1.5	88 : 6	65
3	Zinc(ii) oxide	Tetrachloroethane, 80 °C	1	87 : 10	66
4	Cetylpyridinium bromide	Oxygen, water, 20 °C	0.5	30 : 52	67
5	Copper(ii) oxide	DMF, 20 °C	1	82 : 10	68
6	Sodium dodecyl-sulfate	Water, 20 °C	6	78 : 10	69
7	Iodine	Tetrahydrofuran; water, 20 °C	2	20 : 72	70
8	Zinc(ii) oxide	1,4-Dioxane, 80 °C	1	30 : 69	66
9	Cerium(iii) nitrate hexahydrate	DMF, MW, 100 °C	0.3	69 : 31	71
10	Fe_2_O_3_/silica	Solvent free, 30 °C	8	65 : 22	72
11	Silver NPs	Methanol; water, 55 °C	3	65 : 34	73
12	Aminosulfonic acid	Ethanol, 20 °C	1	55 : 35	74
13	Thiamine hydrochloride	DMF, 20 °C	1.5	42 : 52	65
14	Dodecatungstosilic acid	Ethyl acetate, 20 °C	1.75	43 : 51	75
15	Cerium(iii) nitrate hexahydrate	DMF, 100 °C	4	49 : 51	71
16	Erbium(iii) triflate	Solvent free, 80 °C	0.25	91 : 9	64
17	Silica gel	Solvent free MW	0.1167	7 : 83	76
18	SBA-15/poly(4-styrenesulfonyl(perfluorobutylsulfonyl)imide)	Nitromethane, 25–28 °C	1.3333	65 : 5	77
19	HY zeolite	Acetonitrile, 20 °C	10	61 : 17	78
20	Phosphoric acid	MeOH, 50 °C	0.08	90 : 5	This work

a Reaction conditions: benzaldehyde and *o*-phenylenediamine in various conditions.

b Isolated yield.

## Conclusions

In summary, an efficient protocol for the synthesis of 1,2-disubstituted benzimidazoles *via* a consideration reaction of different aromatic aldehyde (2 mmol) and *o*-phenylenediamine (1 mmol) has been described using phosphoric acid as a homogeneous catalyst. This method is bestowed with several advantages such as a low amount of the catalyst, high isolated yields of pure products, chemical stability of the catalyst, an environmentally benign solvent, an easy experimental workup procedure, and finally the agreement with some of the green chemistry protocols. Moreover, some novel 1,2-disubstituted benzimidazoles as new molecules were synthesized successfully. All the synthesized compounds bind to an agonist in the active site of the 4ll1 protein. These connections lead to the inactivation of this protein and create beneficial effects during the treatment of diabetes. In synthesized compounds, one of the ligands establishes hydrogen bonds with glutamine 107 residues through nitrogens, and in addition, it establishes Π bonds with tyrosine 72. In this study, it was found that these compounds have the potential to become an oral anti-diabetic drug.

## Data availability

The data that supports the findings of this study are available in the ESI† of this article.

## Conflicts of interest

There are no conflicts to declare.

## Supplementary Material

RA-013-D3RA07156A-s001
