# Supplementary material for: Efficient synthesis of 1,2-disubstituted benzimidazoles catalyzed by phosphoric acid as a homogeneous catalyst under mild conditions and investigating their anti-diabetes properties through molecular docking studies and calculations
Source: RSC Adv. 2023 Dec 8;13(51):35781–90. doi: 10.1039/d3ra07156a (PMC10706800; doi:10.1039/d3ra07156a)
Supplement: RA-013-D3RA07156A-s001 [file RA-013-D3RA07156A-s001.pdf]

Electronic Supplementary Material (ESI) for RSC Advances.
This journal is © The Royal Society of Chemistry 2023

Efficient synthesis of 1, 2-disubstituted benzimidazoles catalyzed by phosphoric acid as a homogeneous catalyst under mild conditions and investigating its anti-diabetes properties through molecular docking studies and calculations

Azam Moazeni Bistgani, Abdulhamid Dehghani, Leila Moradi*

Department of Organic Chemistry, Faculty of Chemistry, University of Kashan, Kashan, Iran, P.O. Box 8731753153, +983155912336

Corresponding author, E-mail address: l_moradi@kashanu.ac.ir

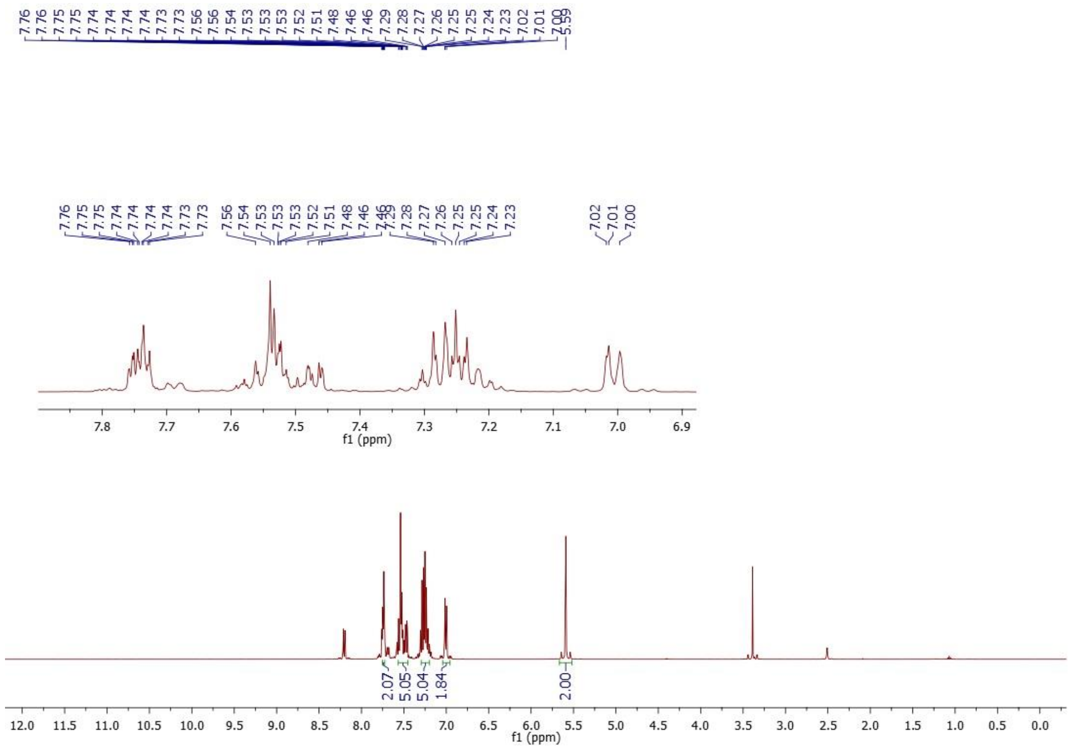

Figure 1*:* ¹H NMR spectrum (400 MHz) of **1-benzyl-2-phenyl-*1*H-benzo[*d*]imidazole (3a)** in DMSO-d_6.

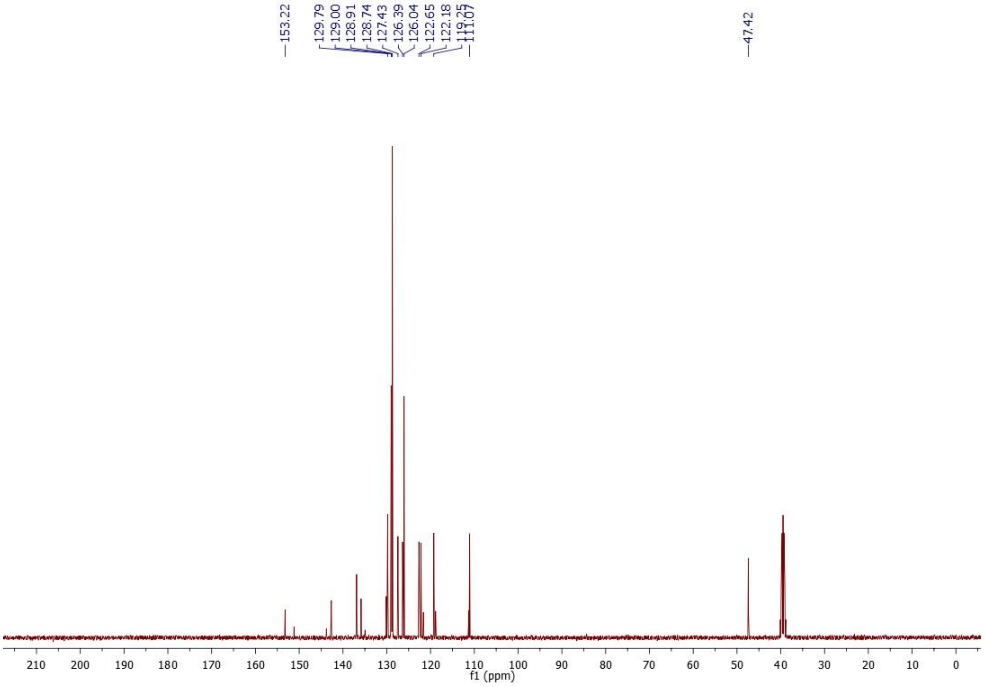

Figure 2*:* ¹³C NMR spectrum (101 MHz) of **1-benzyl-2-phenyl-*1*H-benzo[*d*]imidazole (3a)** in DMSO-d_6.

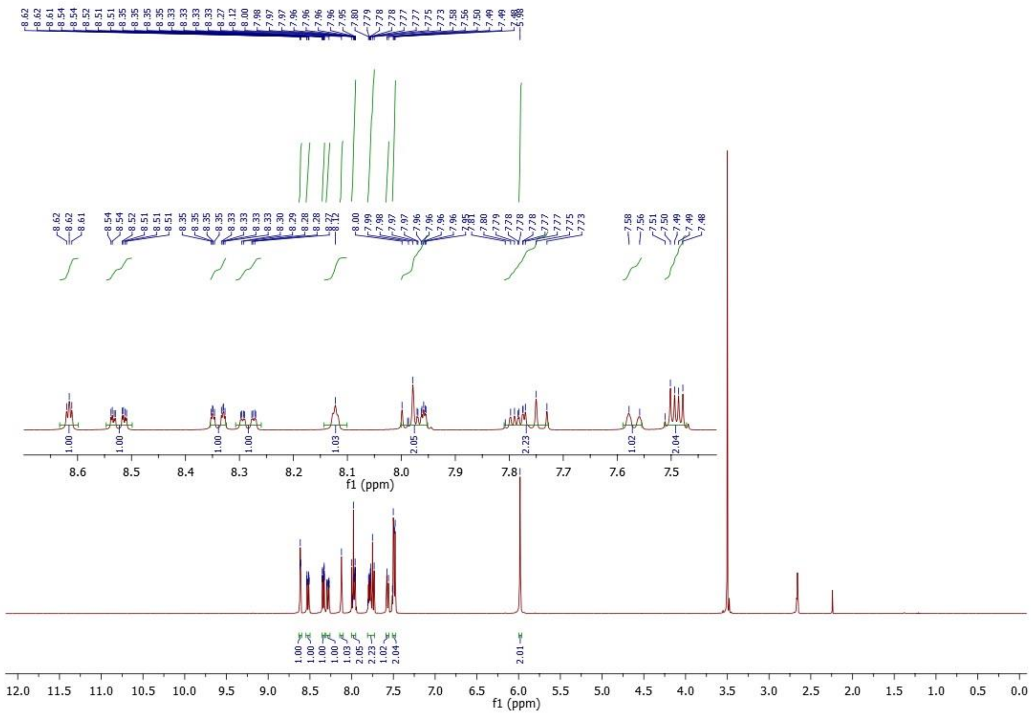

Figure 3*:* ^{1}H NMR spectrum (400 MHz) of **1-(3-nitrobenzyl)-2-(3-nitrophenyl)-1*H*-benzo[*d*]imidazole (3b)** in DMSO-d_6.

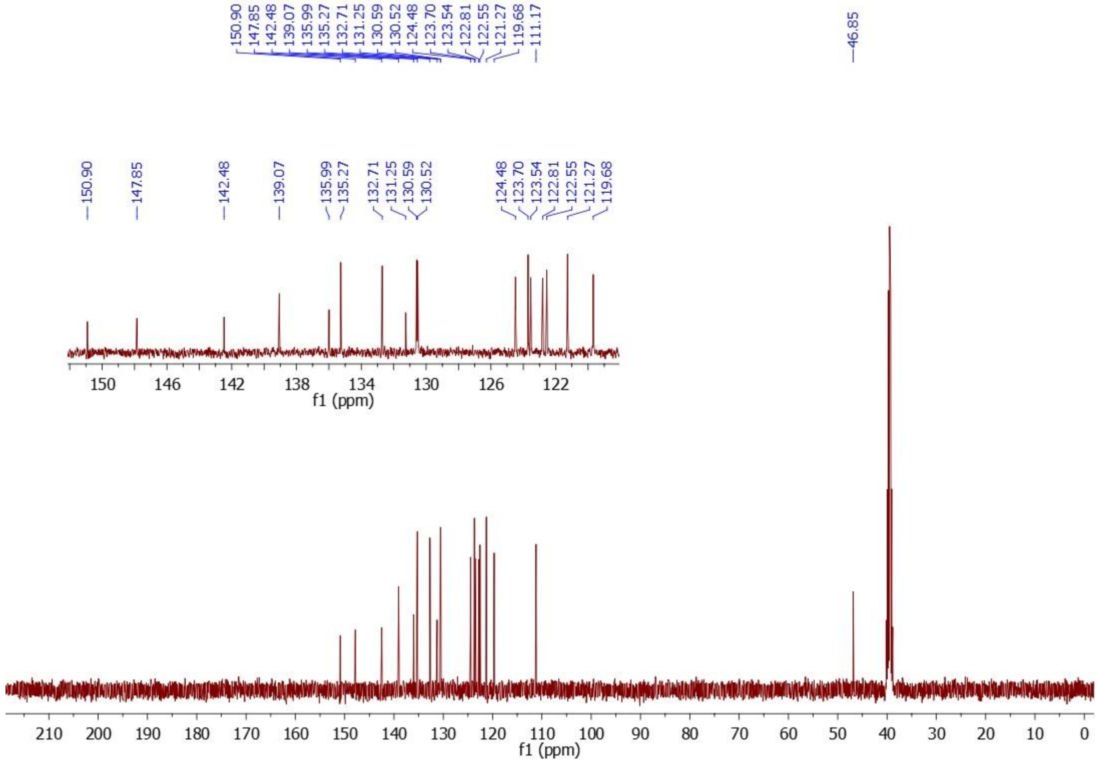

Figure 4*:* ^{13}C NMR spectrum (101 MHz) of **1-(3-nitrobenzyl)-2-(3-nitrophenyl)-1*H*-benzo[*d*]imidazole (3b)** in DMSO-d_6.

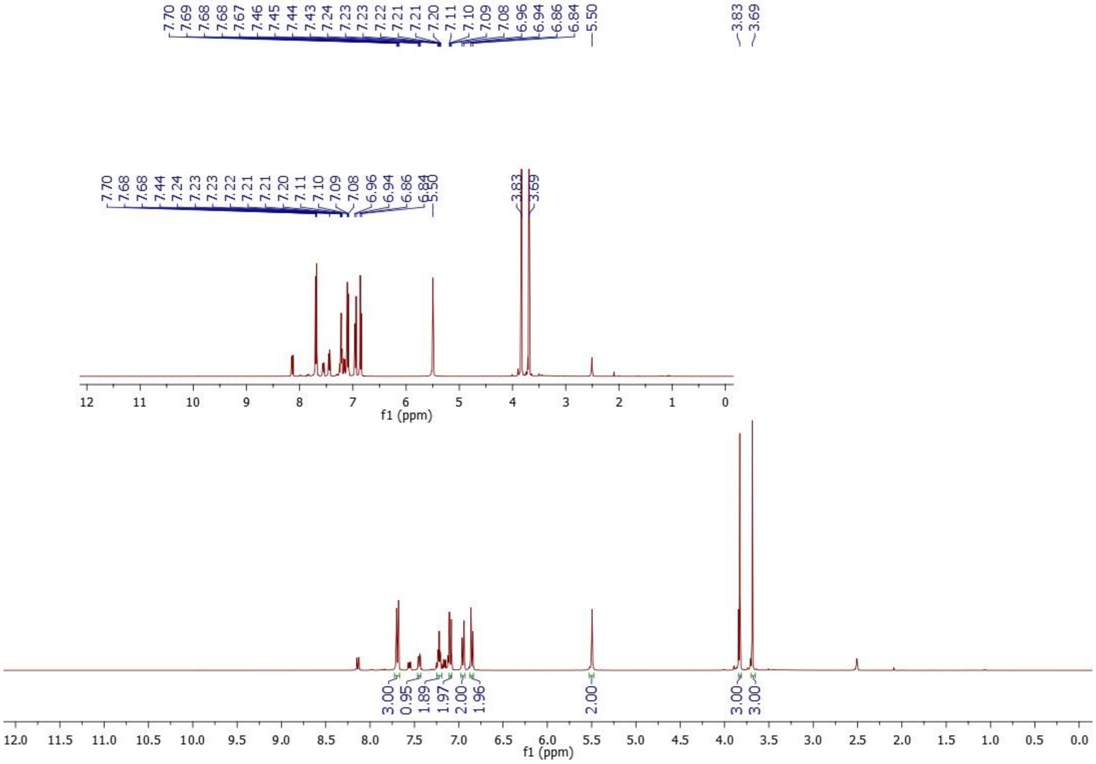

Figure 5*:* ^{1}H NMR spectrum (400 MHz) of **1-(4-methoxybenzyl)-2-(4-methoxyphenyl)-1*H*-benzo[*d*]imidazole (3c)** in DMSO-d_6.

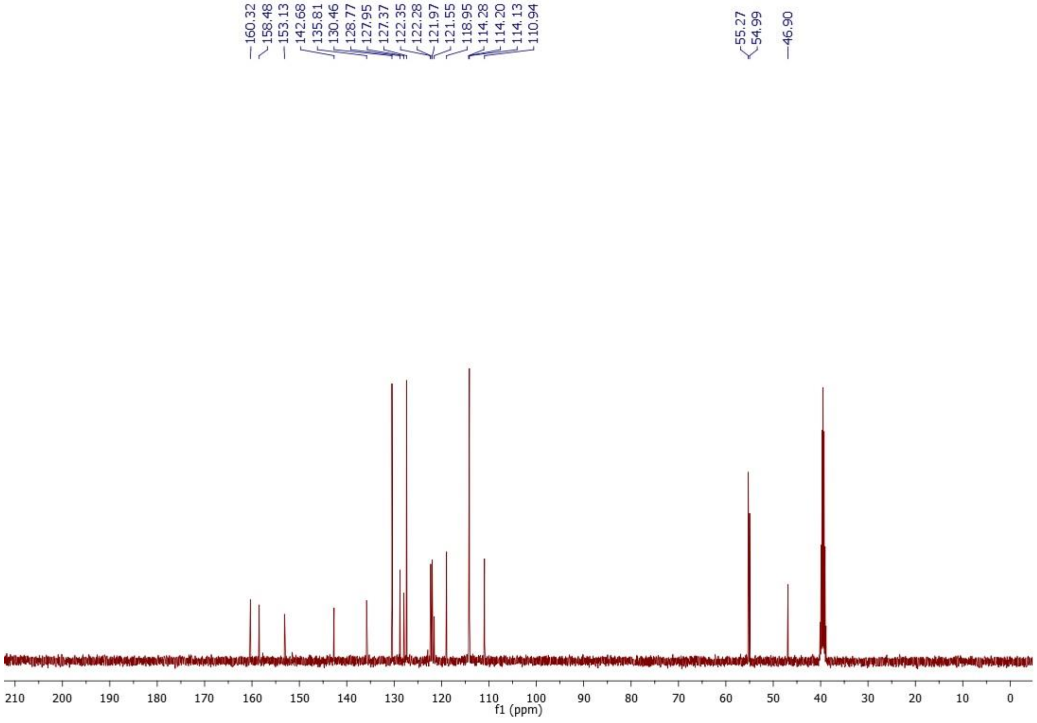

Figure 6*:* ^{13}C NMR spectrum (101 MHz) of **1-(4-methoxybenzyl)-2-(4-methoxyphenyl)-1*H*-benzo[*d*]imidazole (3c)** in DMSO-d_6.

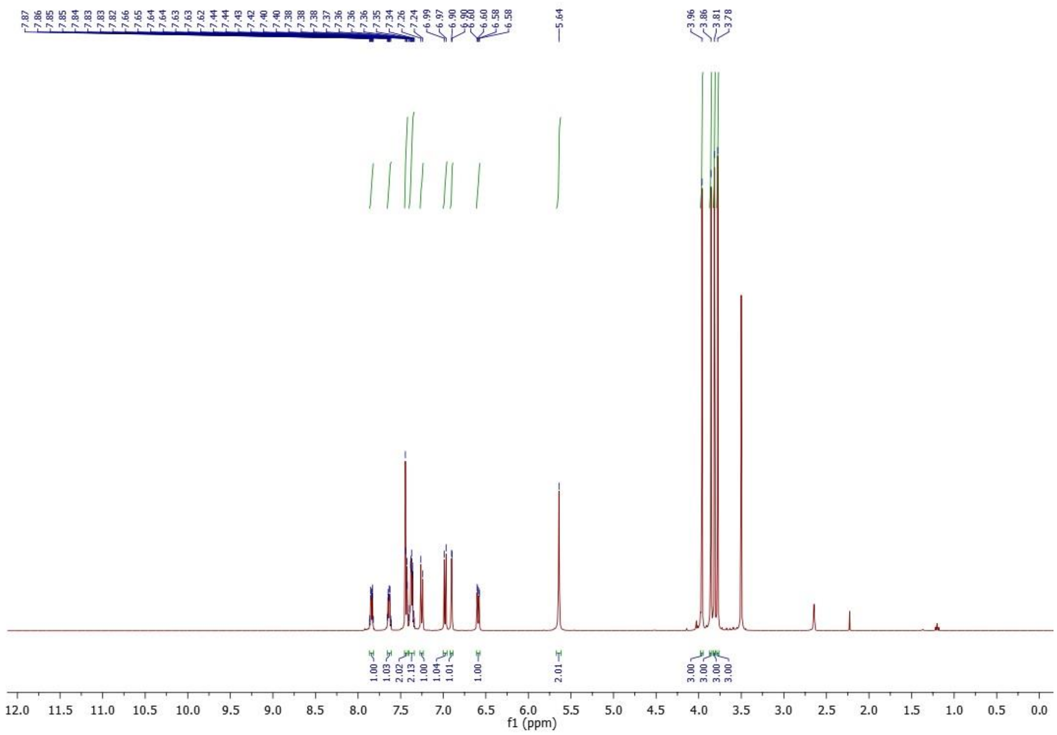

Figure 7: ^{1}H NMR spectrum (400 MHz) of **1-(3,4-dimethoxybenzyl)-2-(3,4-dimethoxyphenyl)-1*H*-benzo[*d*]imidazole (3d)** in DMSO-d_6.

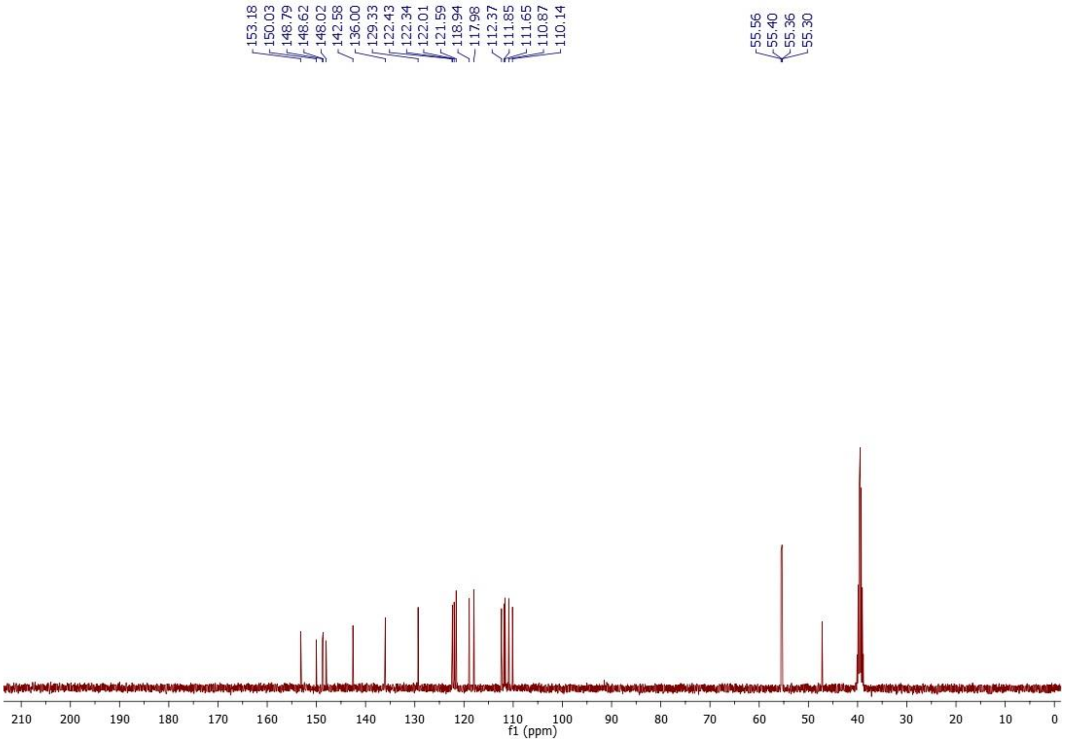

Figure 8: ^{13}C NMR spectrum (101 MHz) of **1-(3,4-dimethoxybenzyl)-2-(3,4-dimethoxyphenyl)-1*H*-benzo[*d*]imidazole (3d)** in DMSO-d_6.

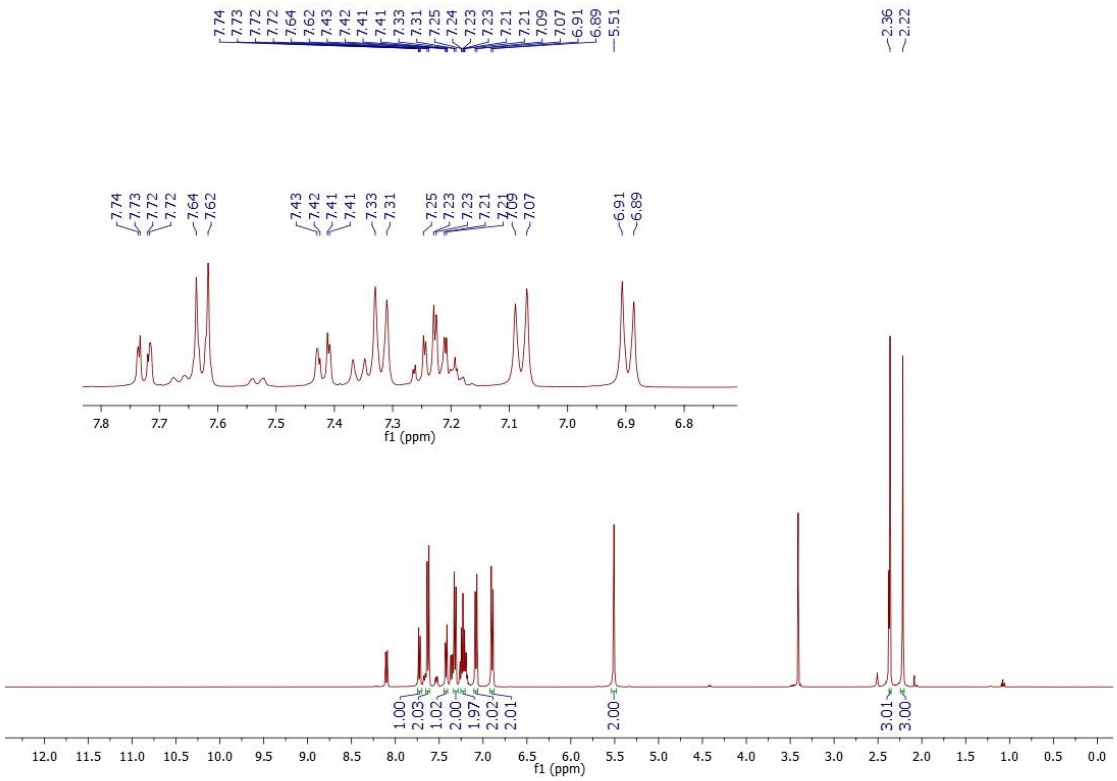

Figure 9*:* ^{1}H NMR spectrum (400 MHz) of **1-(4-methylbenzyl)-2-(p-tolyl)-1*H*-benzo[*d*]imidazole (3e)** in DMSO-d_6.

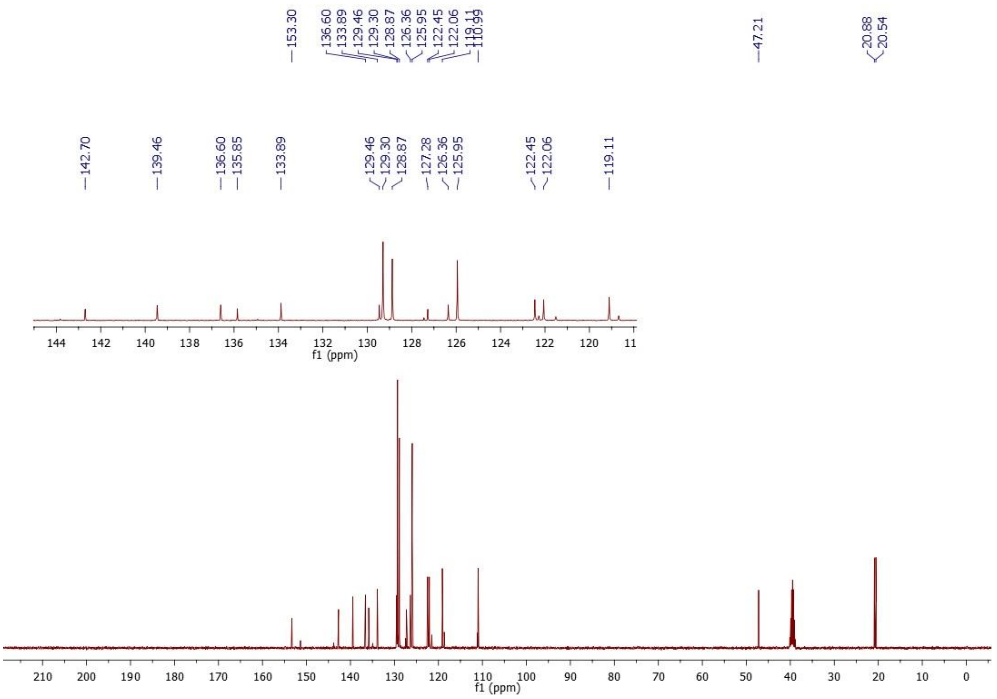

Figure 10*:* ^{13}C NMR spectrum (101 MHz) of **1-(4-methylbenzyl)-2-(p-tolyl)-1*H*-benzo[*d*]imidazole (3e)** in DMSO-d_6.

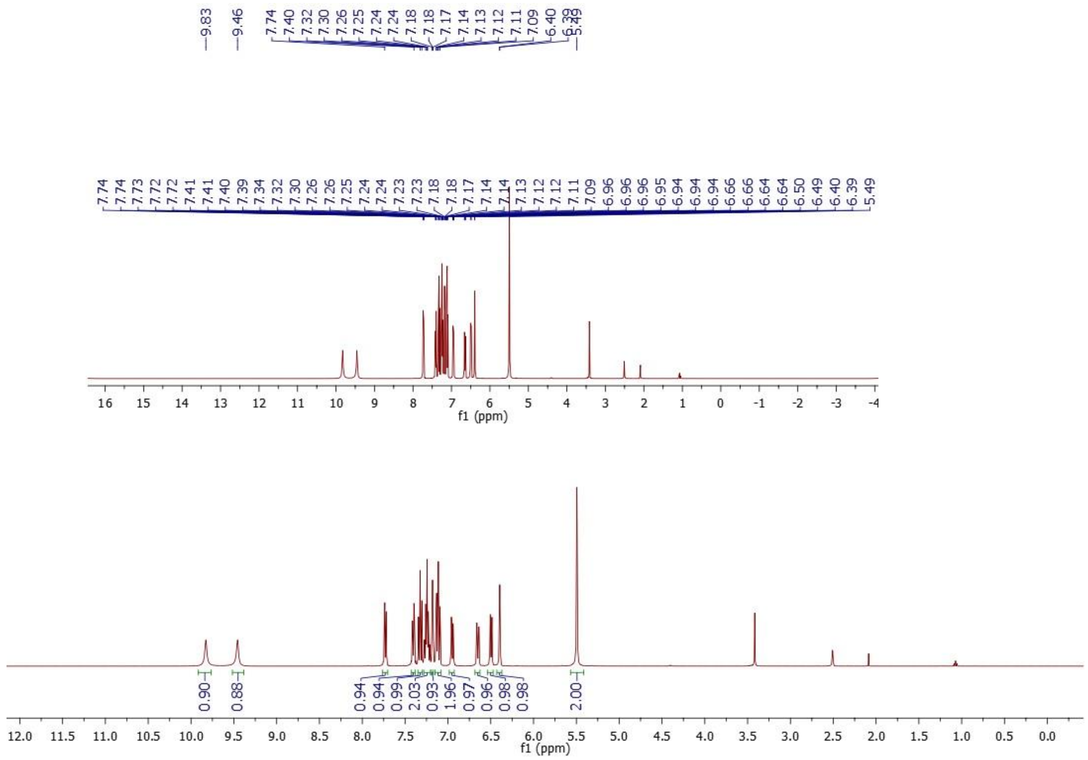

Figure 11*:* ^{1}H NMR spectrum (400 MHz) of **3-(1-(3-hydroxybenzyl)-1H-benzo[d]imidazol-2-yl)phenol (3f)** in DMSO-d_6.

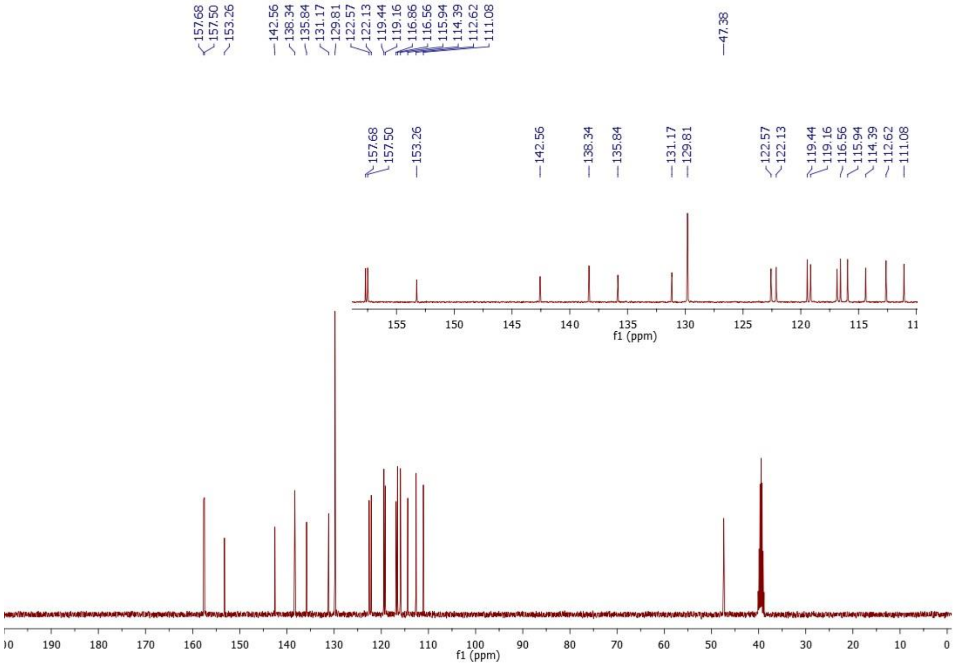

Figure 12*:* ^{13}C NMR spectrum (400 MHz) of **3-(1-(3-hydroxybenzyl)-1H-benzo[d]imidazol-2-yl)phenol (3f)** in DMSO-d_6.

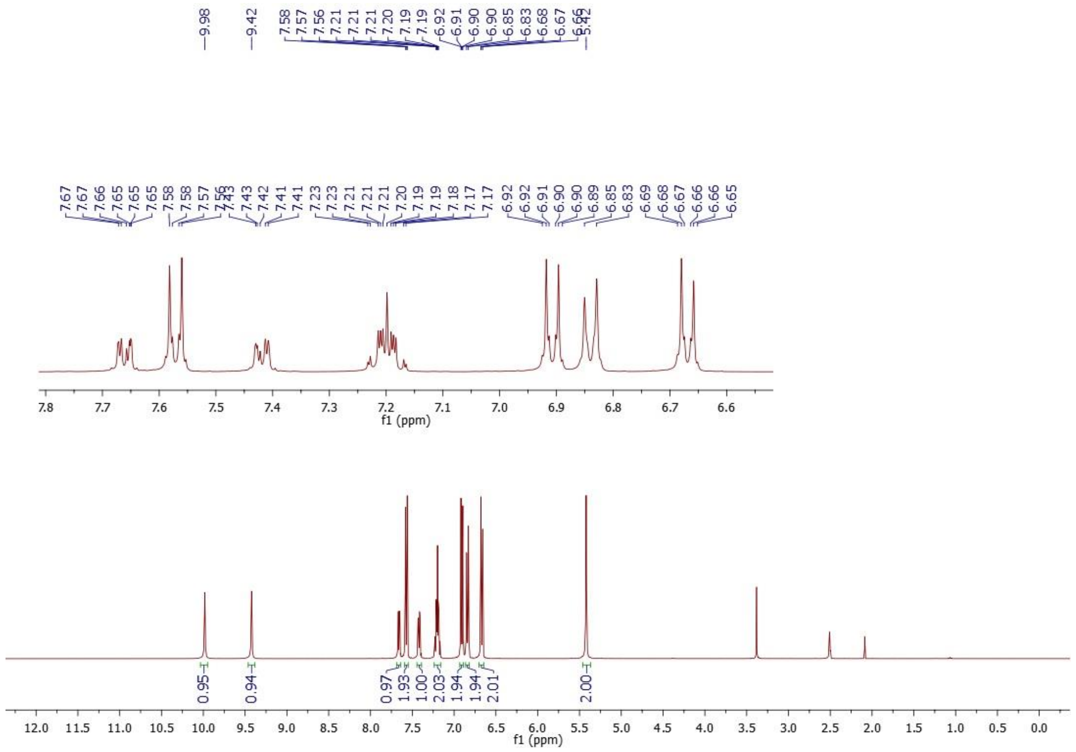

Figure 13*:* ^{1}H NMR spectrum (400 MHz) of **4-(1-(4-hydroxybenzyl)-1*H*-benzo[*d*]imidazol-2-yl)phenol (3g)** in DMSO-d_6.

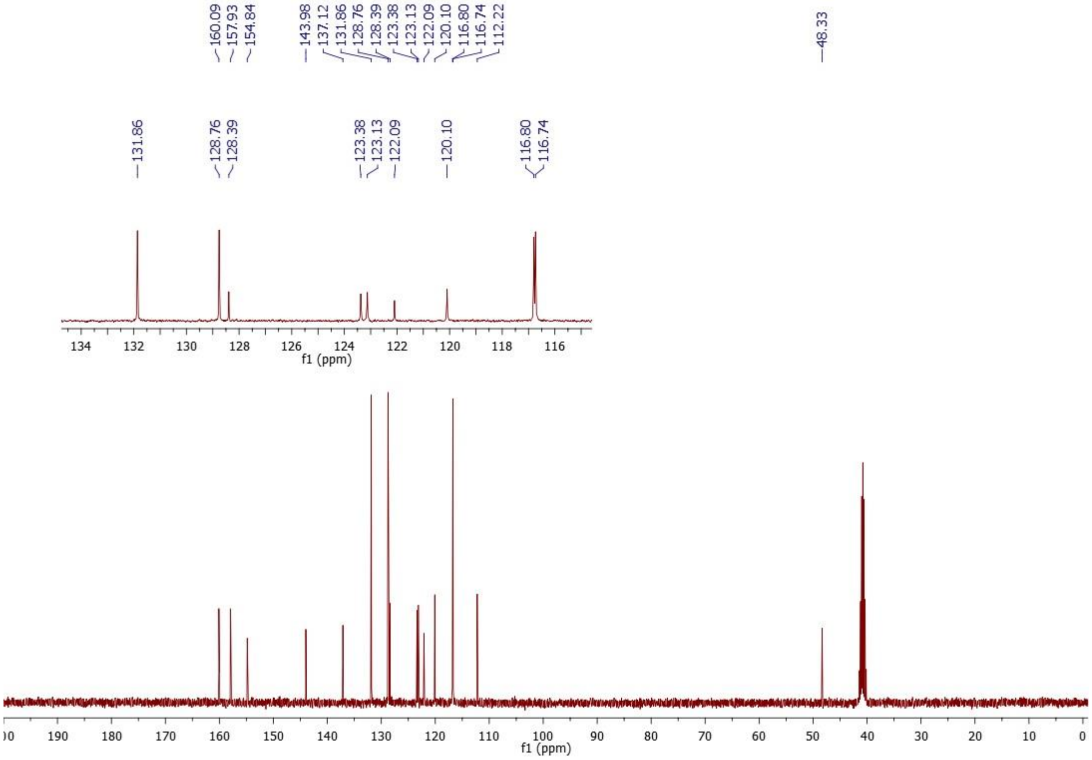

Figure 14*:* ^{13}C NMR spectrum (101 MHz) of **4-(1-(4-hydroxybenzyl)-1*H*-benzo[*d*]imidazol-2-yl)phenol (3g)** in DMSO-d_6.

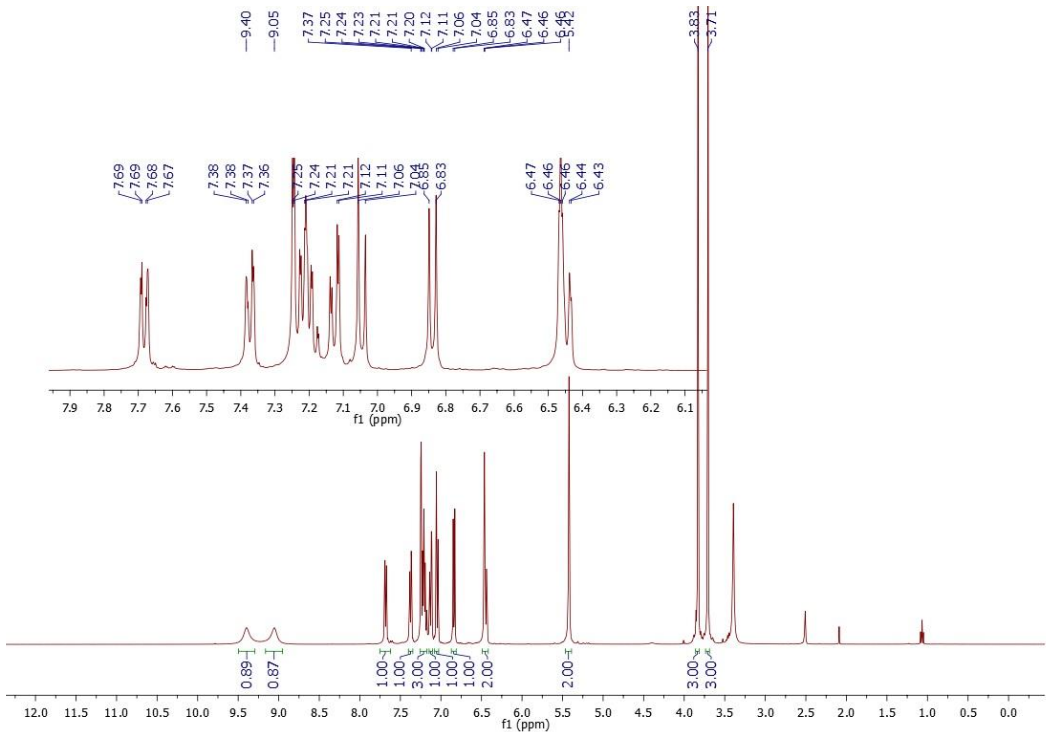

Figure 15: ^{1}H NMR spectrum (400 MHz) of **5-(1-(3-hydroxy-4-methoxybenzyl)-1*H*-benzo[*d*]imidazol-2-yl)-2-methoxyphenol (3h)** in DMSO-d_6.

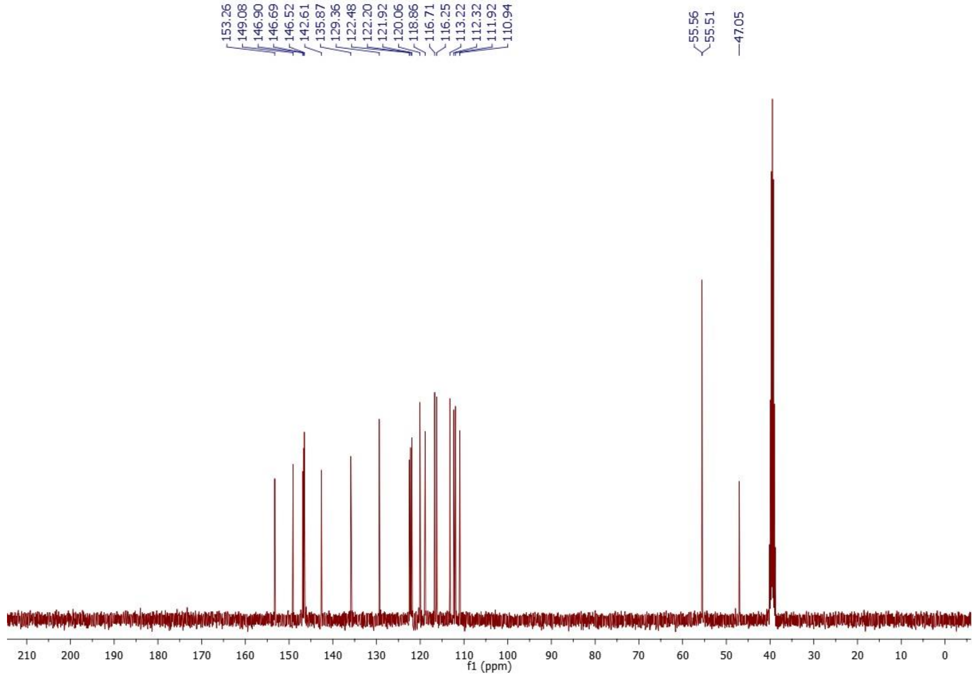

Figure 16: ^{13}C NMR spectrum (101 MHz) of **5-(1-(3-hydroxy-4-methoxybenzyl)-1*H*-benzo[*d*]imidazol-2-yl)-2-methoxyphenol (3h)** in DMSO-d_6.

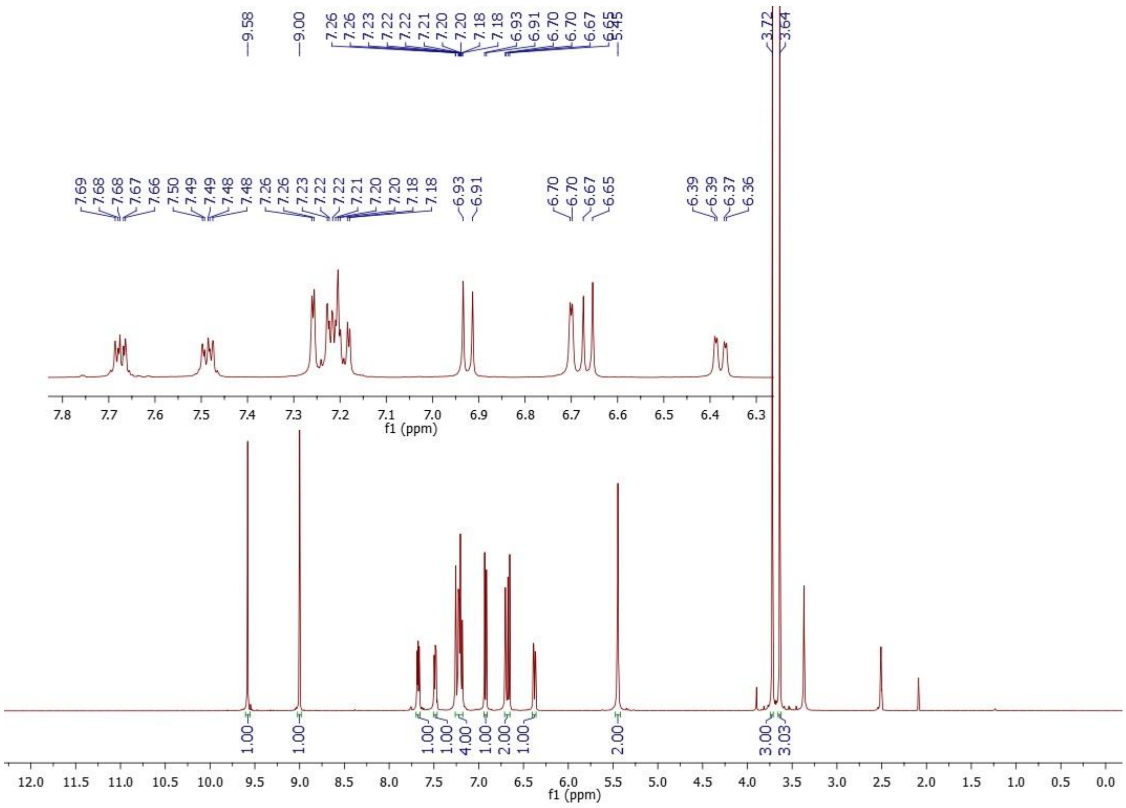

Figure 17: ^{1}H NMR spectrum (400 MHz) of **4-(1-(4-hydroxy-3-methoxybenzyl)-1*H*-benzo[*d*]imidazol-2-yl)-2-methoxyphenol (3i)** in DMSO-d_6.

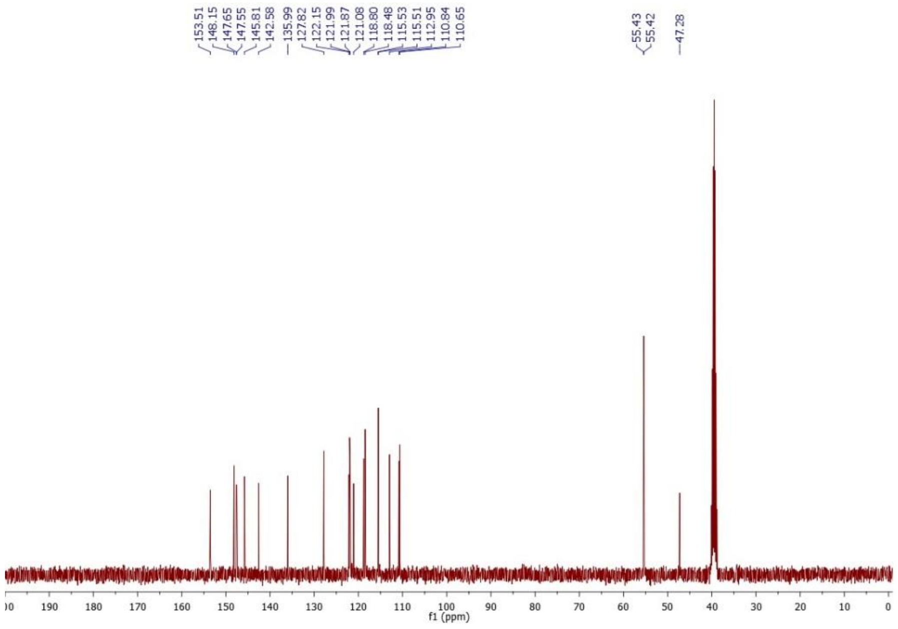

Figure 18: ^{13}C NMR spectrum (101 MHz) of **4-(1-(4-hydroxy-3-methoxybenzyl)-1*H*-benzo[*d*]imidazol-2-yl)-2-methoxyphenol (3i)** in DMSO-d_6.

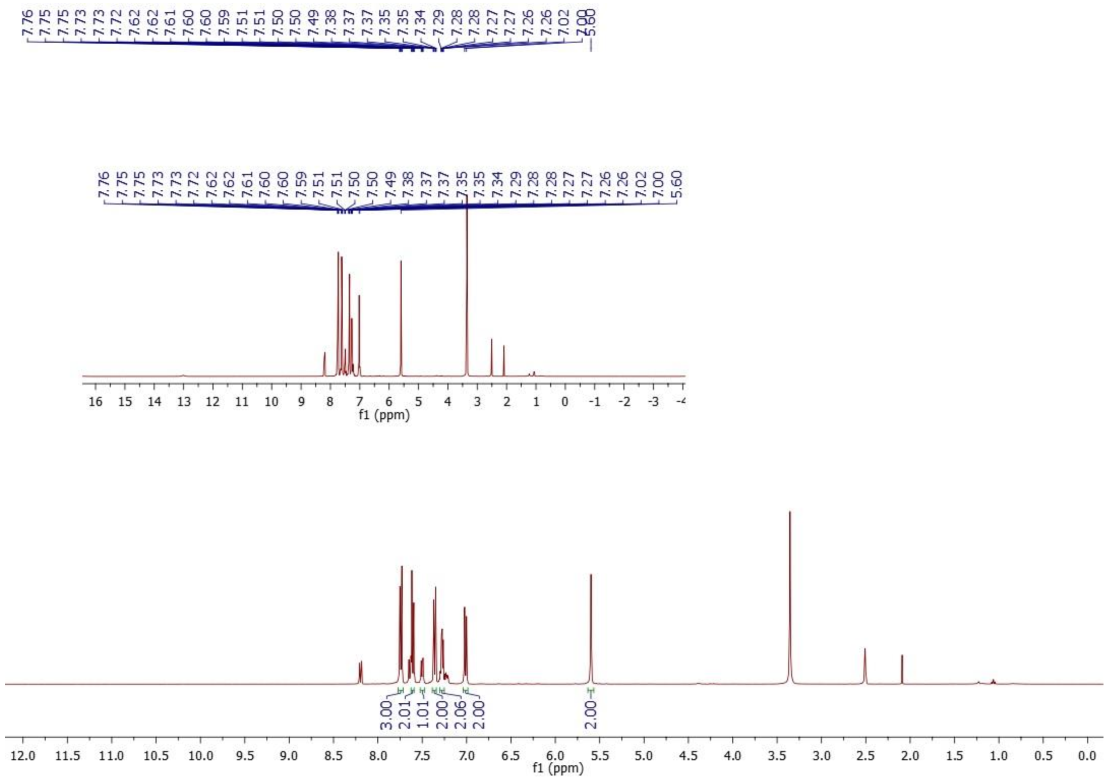

Figure 19*:* ^{1}H NMR spectrum (400 MHz) of **1-(4-chlorobenzyl)-2-(4-chlorophenyl)-1*H*-benzo[*d*]imidazole (3j)** in DMSO-d_6.

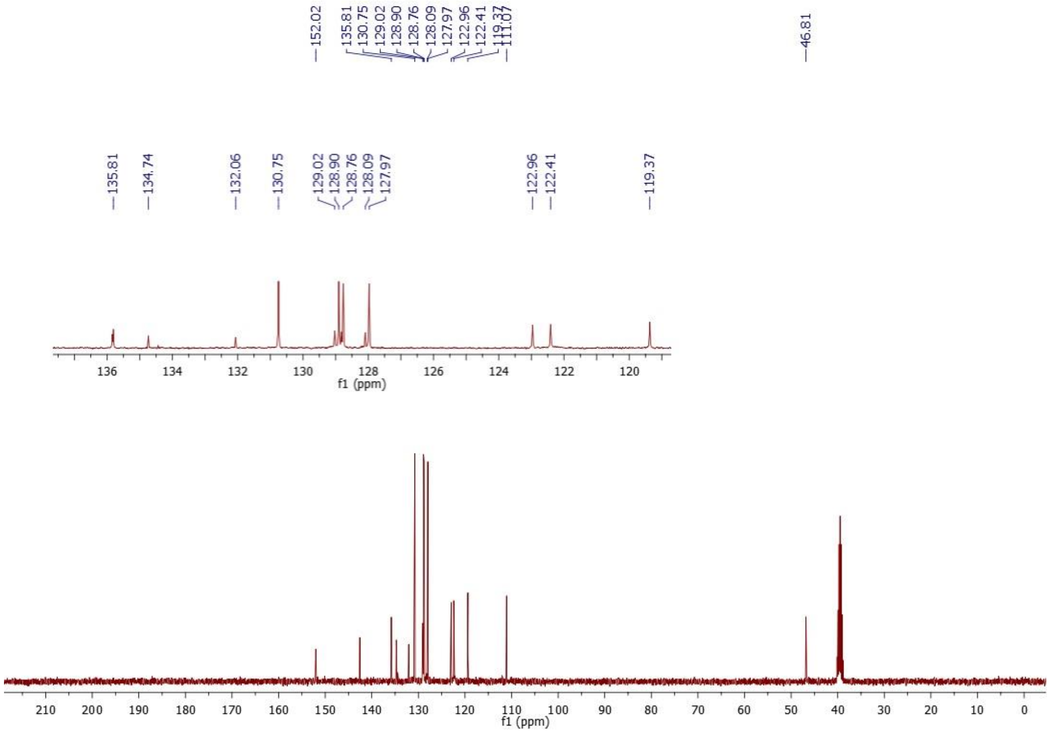

Figure 20*:* ^{13}C NMR spectrum (101 MHz) of **1-(4-chlorobenzyl)-2-(4-chlorophenyl)-1*H*-benzo[*d*]imidazole (3j)** in DMSO-d_6.

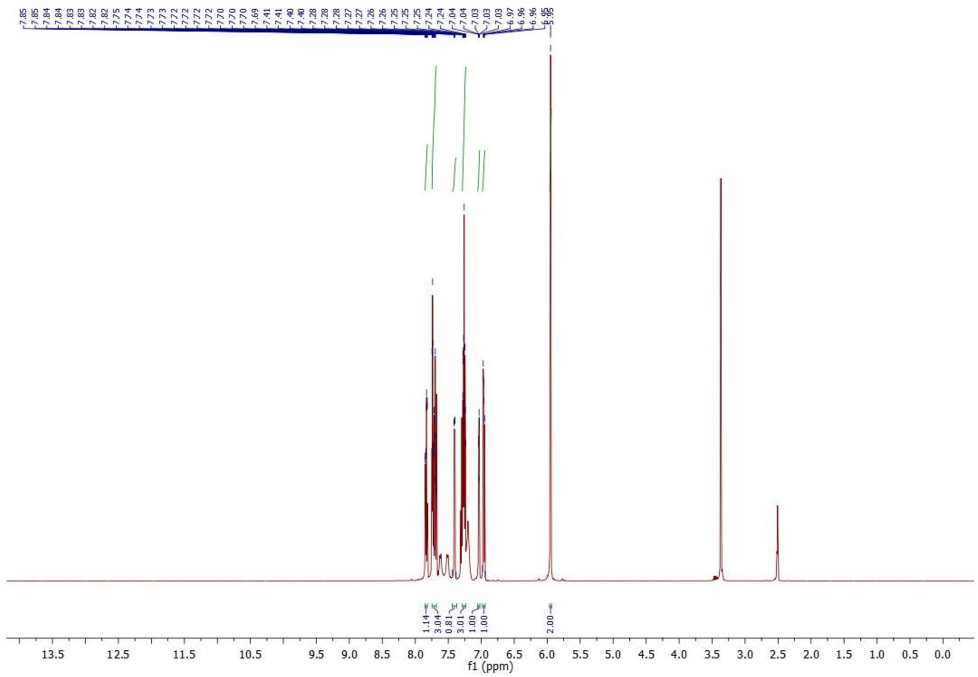

Figure 21*:* ^{1}H NMR spectrum (400 MHz) of **2-(thiophen-2-yl)-1-(thiophen-2-ylmethyl)-1H-benzo[d]imidazole (3k)** in DMSO-d_6.

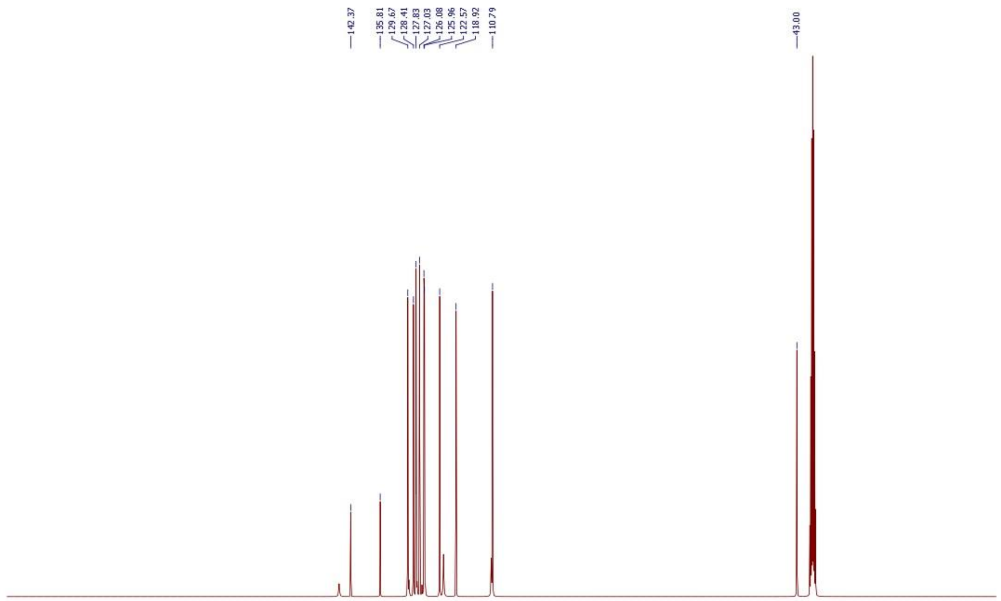

Figure 22*:* ^{13}C NMR spectrum (101 MHz) of **2-(thiophen-2-yl)-1-(thiophen-2-ylmethyl)-1H-benzo[d]imidazole (3k)** in DMSO-d_6.
